# Transfer of Spatial Contact Information Among Limbs and the Notion of Peripersonal Space in Insects

**DOI:** 10.3389/fncom.2018.00101

**Published:** 2018-12-18

**Authors:** Volker Dürr, Malte Schilling

**Affiliations:** ^1^Biological Cybernetics, Faculty of Biology, Bielefeld University, Bielefeld, Germany; ^2^Cluster of Excellence Cognitive Interactive Technology (CITEC), Bielefeld University, Bielefeld, Germany

**Keywords:** affordance, spatial coordination, limb movement, touch, peripersonal space, stick insect, whole-body kinematics, artificial neural network

## Abstract

Internal representation of far-range space in insects is well established, as it is necessary for navigation behavior. Although it is likely that insects also have an internal representation of near-range space, the behavioral evidence for the latter is much less evident. Here, we estimate the size and shape of the spatial equivalent of a near-range representation that is constituted by somatosensory sampling events. To do so, we use a large set of experimental whole-body motion capture data on unrestrained walking, climbing and searching behavior in stick insects of the species *Carausius morosus* to delineate ‘action volumes’ and ‘contact volumes’ for both antennae and all six legs. As these volumes are derived from recorded sampling events, they comprise a volume equivalent to a representation of coinciding somatosensory and motor activity. Accordingly, we define this volume as the *peripersonal space* of an insect. It is of immediate behavioral relevance, because it comprises all potential external object locations within the action range of the body. In a next step, we introduce the notion of an *affordance space* as that part of peripersonal space within which contact-induced spatial estimates lie within the action ranges of more than one limb. Because the action volumes of limbs overlap in this affordance space, spatial information from one limb can be used to control the movement of another limb. Thus, it gives rise to an affordance as known for contact-induced reaching movements and spatial coordination of footfall patterns in stick insects. Finally, we probe the computational properties of the experimentally derived affordance space for pairs of neighboring legs. This is done by use of artificial neural networks that map the posture of one leg into a target posture of another leg with identical foot position.

## Introduction

Like humans, animals have internal representations of space (Jeffery, 2003). In humans, internal representations of space have been categorized in conjunction with distinct spatial volumes, which correspond to different sensory cues about the ambient space, often with correspondingly distinct neuronal substrates (for review see Previc, 1998; Holmes and Spence, 2004). Such representations directly sub-serve behavior and play a functional role as internal models in control of goal-directed movements in humans (Kawato, 1999) and in robot motor control (Schillaci et al., 2016). In particular, peripersonal space is defined as a near-range area on which humans can directly act, i.e., which is “within reach.” While there is considerable debate about how sharp the boundary of human peripersonal space is (Bufacchi and Iannetti, 2018), there is agreement on that it differs functionally from the space further away and is connected to specific neuronal substrates in parietal and premotor areas (e.g., Cléry and Hamed, 2018).

Whereas, in non-primate mammals and, potentially, other vertebrate groups such as birds, the existence of homologous neuronal substrates suggest the existence of similar, multiple internal representations of space as in humans, the situation is much less clear in invertebrates. One reason for this may be the conceptual problem that the distinction of internal representations of space must be linked to behavioral performance, for example as distinct skills or differential use of spatial cues related to different spatial volumes. In insects, at least two kinds of spatially coordinated behavior can be discerned that, most likely, are linked to distinct internal models: The first of these concerns the spatially coordinated movement of limbs and body parts, for example during locomotion on or manipulation of the near-range environment. A corresponding internal representation of near-range space is required whenever spatial information has to be shared by multiple body parts. Potential neural substrates of internal near-range representations are topological afferent projections such as those described for the cricket cercal system (Jacobs et al., 2008) or for mechanoreceptor afferents of locust legs (e.g., Mücke and Lakes-Harlan, 1995; Newland et al., 2000). A recent systematic inventory of somatosensory projections in fruit flies suggests parallels to the somatosensory system of mammals (Tsubouchi et al., 2017). The second type of spatially coordinated behavior concerns course control and navigation in far-range space, i.e., space beyond the immediate action range of the limbs and body parts. In insects, the spatial representation of far-range cues has been studied intensely in the context of visually guided locomotion. An example is the self-motion dependent modulation of visual interneurons (Chiappe et al., 2010) that gives rise to a representation of walking direction in the optic lobes of walking fruit flies (Fujiwara et al., 2017). Also, the central complex is well known to be involved in behaviors relying on estimates of distance and direction. Prominent examples include the encoding of celestial direction cues in locusts (Heinze and Homberg, 2007) and of heading direction in walking fruit flies (Green et al., 2017; Turner-Evans et al., 2017).

Thus, with regard to behavioral relevance of spatial sensory cues, an obvious boundary is defined by the volume that is “within reach” of any body part, the limbs in particular. This is plausible because sensory modalities such as touch or taste depend on contact cues on the body surface and therefore cannot be experienced beyond the spatial range spanned by all possible movements of the body trunk, and limbs. In contrast, vision, audition and smell transduce the energy from photons, sound pressure waves or volatile chemicals, most of which typically originate from locations beyond the own body. They are “beyond reach.” The present study combines behavioral and computational considerations about the spatial volume “within reach” in walking and climbing insects. We will argue that this is in many ways equivalent to what is called peripersonal space in humans. The spatial volume “within reach” of the human body is perceived in a way that relates our ability to act and interact within that spatial volume. In order to capture this, internal models must be grounded in sensorimotor representations that relate body posture and movement to the corresponding part of space. At their core, internal models reflect functional, modular organization of the body (Davidson and Wolpert, 2004; Cothros et al., 2006) with redundancy. As an example, Patané et al. (2017) showed a dissociation between peripersonal and interpersonal space which they found to be largely overlapping, though clearly dissociable: the peripersonal space being delimited as the space reachable with a tool. Other hallmarks of human internal models are their flexibility, e.g., in case of tool use (Cardinali et al., 2009) and their multimodal organization, e.g., when estimating hand position from somatosensory, proprioceptive, visual and even auditory information (Makin et al., 2008). Despite its multimodal nature, most experimental work on human peripersonal space has focused on vision, often in relation to eye-hand coordination. However, since peripersonal space occurs in congenitally blind humans (Ricciardi et al., 2017), it must develop independently of vision. Ricciardi et al. suggested that, therefore, internal models in humans directly relate to the configurations of limbs relative to each other, thus forming an internal body model.

Whether or not insects may have an internal body model with similar properties to those in humans is unknown. It is clear, however, that insects readily climb about in spatially cluttered environment, thus demonstrating their ability of flexible and reliable spatial coordination of a multi-limbed body with many degrees of freedom. An important component of this ability is the transfer of spatial information from one limb to another. Essentially, this transfer turns the spatial knowledge acquired by one limb into an affordance for another limb. For example, the physical contact of one limb with an obstacle may be used to guide the movement of another limb, in order to exploit prior knowledge about foothold/grip locations and to achieve contact at a nearby location. Our use of the term affordance follows the definition by J. J. Gibson, as a behavioral option of an animal that is signaled by a combination of sensory features (Gibson, 1977, p. 79: “*an affordance […] is a combination of physical properties of the environment that is uniquely suited to a given animal – [… e.g. its] locomotor system*.”). Behavioral evidence suggests that spatial coordination of limbs in insects ranges from pre-programmed, open-loop behaviors, to closed-loop control of limb posture, and to complex coordinate transfer among neighboring limbs. For example, grooming movements are often considered pre-programmed rhythmical limb movements, as in eye-cleaning behavior of the cricket (Honegger et al., 1979), or in grooming of various body locations in locusts (Berkowitz and Laurent, 1996) and fruit flies (Seeds et al., 2014). At least in the case of locusts, so-called grooming movements of the forewing have been shown to form a continuum of movements (Dürr and Matheson, 2003), consistent with the idea of a continuous encoding of the wing surface location by an array of mechanoreceptors (Page and Matheson, 2004). Although the neuronal substrate underlying these aimed limb movements are largely unknown until today, within-trial adjustment of limb posture suggests that they are subject to feedback control (see Figure 6 in Matheson, 1998) and plasticity of proprioceptive encoding of limb posture proves that the corresponding neural representation is adaptive (Page and Matheson, 2009).

Regarding coordinate transformation among limbs, several studies have demonstrated this to occur in stick insects, including standing (Cruse, 1979), walking (Dean and Wendler, 1983) and climbing animals (Theunissen et al., 2014). Targeting behavior of legs has been transferred into models of motor control. These demonstrate qualitatively how such mappings can be realized using a local transformation (Dean et al., 1999) or, in the case of more complex walking behavior, by applying an internal body model (Schilling and Cruse, 2012). In stick insects, the ipsilateral transfer of postural cues not only works between pairs of walking legs, but also between the antennae and front legs (Schütz and Dürr, 2011). In the latter case, antennal contact cues can elicit fast re-targeting of on-going swing movements, effectively turning a swing movement into an aimed reach-to-grasp movement of a front leg (for review, see Dürr et al., 2018). Visual estimates of distance “within reach” have been shown to occur in gap crossing behavior in fruit flies (Pick and Strauss, 2005), suggesting that these insects also have a reliable estimate of their own body size and/or action range (Strauss et al., 2011; Krause, 2015). Visually mediated coordinate transformations allow for targeted front leg movements in locusts (Niven et al., 2010) and horse-head grasshoppers (Niven et al., 2012). In this kind of behavior, locusts combine monocular visual inputs with mechanosensory inputs from their antennae before the onset of a step, i.e., during motion planning. Similar to spatially targeted grooming movements as mentioned above, visually induced reaching in locusts requires proprioceptive sensory information from the femoral chordotonal organ. Finally, a very fast, ballistic, visually induced type of leg movement is the front leg strike of praying mantises (Maldonado et al., 1967; Corrette, 1990) and mantispids (Kral et al., 2000) that strike to catch prey.

Given this body of evidence on spatially targeted limb movements, their plasticity and multimodal control, we claim that the insect body is surrounded by an ambient volume that is functionally equivalent to peripersonal space in humans. With particular reference to the coordinate transfer among limbs in stick insects, we suggest that the peripersonal space in insects may be defined by the shared use of spatial information among two or more body parts. Accordingly, the objectives of this study are (i) to determine the size, shape and locations of action volumes from whole-body motion capture data on unrestrained climbing stick insects; (ii) to investigate the relative size of contact volumes, i.e., the regions where contacts are particularly likely to occur during natural locomotion; and (iii) to determine the size and shape of affordance volumes, i.e., the overlap of contact volumes of pairs of limbs. In our case, a contact event at one limb, together with the corresponding proprioceptive information about the posture of this limb, generates the behavioral option for another limb to reach for the contact location. The underlying coordinate transformation is a basic functional property of motor control systems in limbed animals in general. Therefore, our final objective is to (iv) understand the computational complexity of such transformations in an insect. Using artificial neural network models of different complexities we assess the performance of the reciprocal spatial mappings among pairs of legs that share an affordance volume. By. doing so, we provide a basic notion of an internal model for near-range space in insects. This may serve as a computational ground plan for spatial coordination in other limbed animals.

## Materials and Methods

### Experimental Data Set

**Figure 1:**
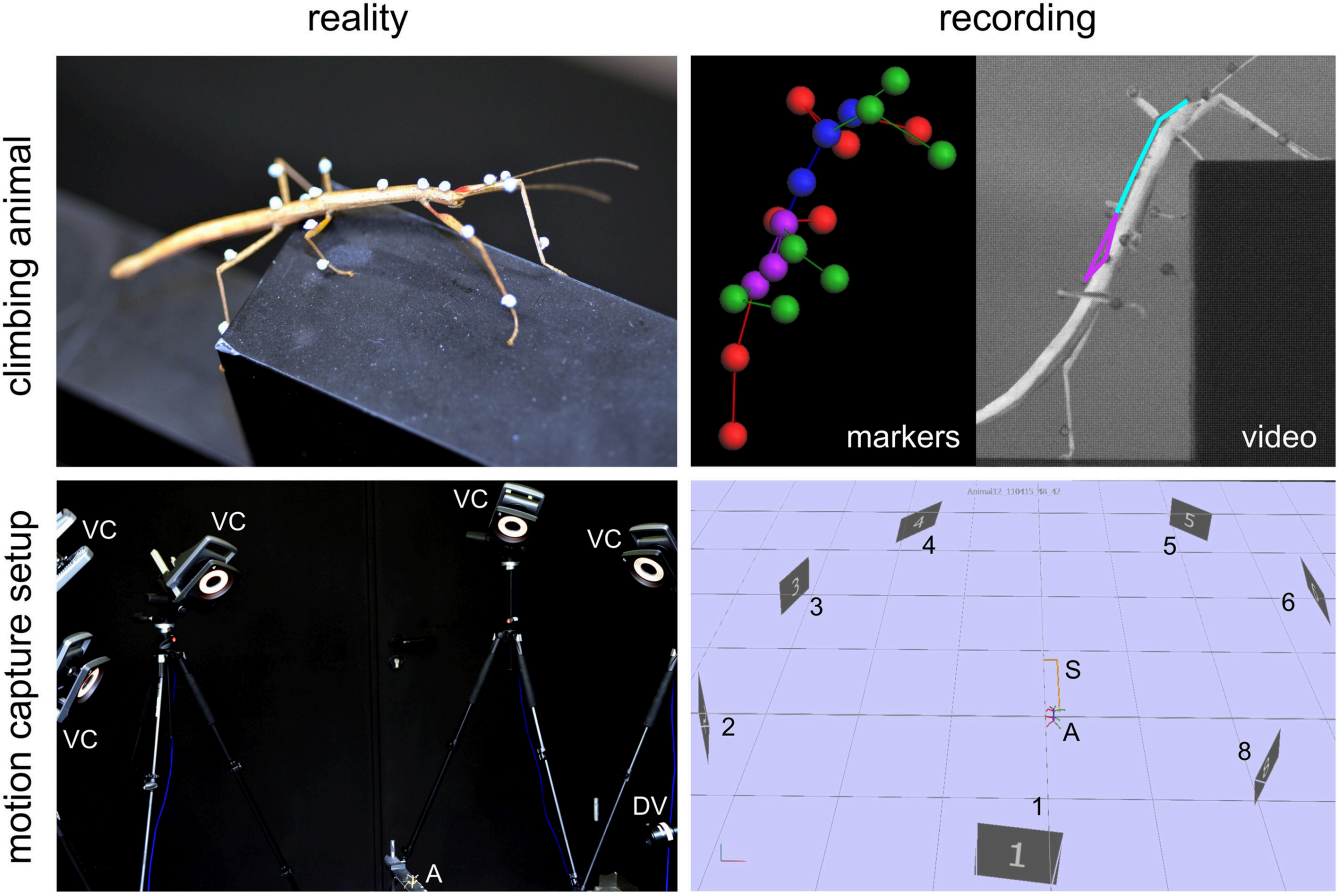
Motion capture setup and recording. Photographs (**left column**) and software screenshots (**right column**) of a recorded stick insect **(top row)** and the motion capture system (**bottom row**). Animals (A) were labeled with small retro-reflective markers and their whole-body kinematics recorded by means of a marker-based motion capture system with eight Vicon cameras (VC, numbered 1–8 in right bottom panel) and an additional digital video camera (DV). The motion capture data yielded sets of labeled marker trajectories (top right panel: markers) that allowed geometrical reconstruction and kinematic analysis of the animal posture (top right panel: video) in 200 frames per second. Note that the setup (S) shown here was only one of three variants used in this study.

All experimental data used in this study were acquired in behavioral experiments on unrestrained walking and climbing, adult, female stick insects of the species *Carausius morosus* (de Sinéty, 1901). Animals were bred at the animal facility of the Biological Cybernetics Department of Bielefeld University, where they were kept in a 12:12 h light:dark cycle and room temperature around 24°C. All data used for the calculation of spatial volumes were acquired with a marker-based motion capture system (Vicon MX10 equipped with eight T10 cameras, Figure 1) as described by Theunissen and Dürr (2013). Temporal resolution was 200 frames per second and spatial precision of the 3D marker position measurements was approximately 0.1 mm. Three different types of setups were used to record a variety of walking, climbing and searching movements of the legs and the antennae. In all cases, the animals walked along a flat horizontal walkway that was 40 mm wide.

**Figure 2:**
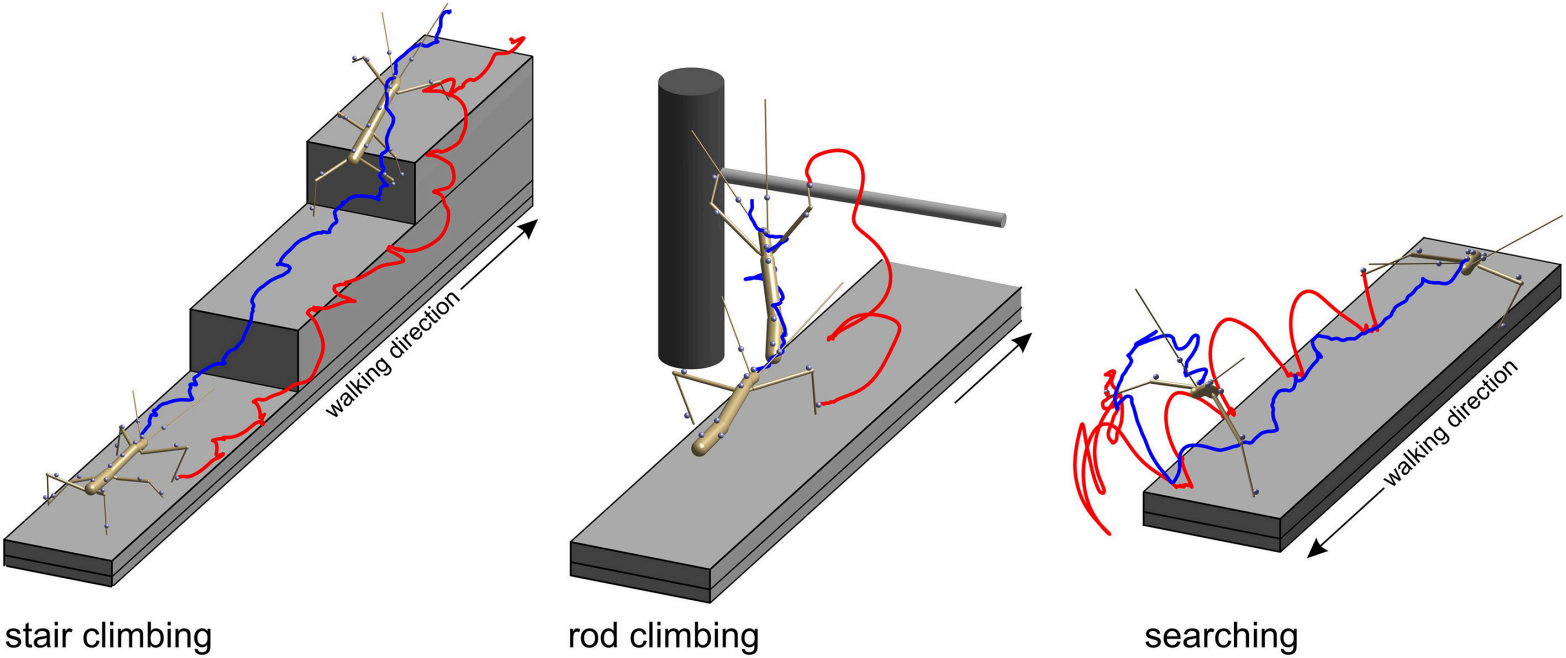
Three types of setups were used to acquire experimental data. In all paradigms, stick insects were motion-captured as they walked along a 40 mm wide walkway. Two recorded postures are shown, one at the beginning of the trial and another near the end of the trial. Gray spheres show marker locations. Only the tracked body segments are shown. Colored lines show the trajectories of the tibia-tarsus joint of the right front leg (red) and of the head (blue). Left: In the *stair-climbing paradigm* the animals encountered two stairs of different height (here 24 mm) which they climbed readily. In trials of this paradigm all legs and thorax segments were recorded. In some trials, also the head and antennae were recorded. Middle: in the *rod-climbing* paradigm the animals encountered a horizontal rod held across the walkway at different height. In trials of this paradigm, only the antennae and front legs were recorded, along with the head and thorax segments. Right: In the *searching paradigm*, animals stepped across the far edge of the walkway and engaged in rhythmic searching movement of the antennae and front legs. In trials of this paradigm, only the antennae and front legs were recorded, along with the head and prothorax.

In the *stair-climbing setup*, a set of two stairs was placed on the distal third of the walkway (Figure 2, left). The stairs were of different height (8, 24, or 48 mm), so that animals had to adapt their climbing behavior to different obstacles, resulting in height-dependent changes in body inclination (Theunissen et al., 2015) or the relative frequency of short correction steps (Theunissen and Dürr, 2013). A flat walkway was used as reference condition. A total of 365 stair-climbing trials from ten animals were included in the present analysis. In each trial, motion capture analysis yielded the joint position and joint angle time courses of all six legs, along with the position time courses of all segment boundaries of the thorax and the head. Thirty-four trials of one animal also comprised the joint angle and tip position time courses of both antennae. This stair-climbing data have been used before in original research publications on distinct step types (Theunissen and Dürr, 2013), spatial coordination of foot contacts (Theunissen et al., 2014) and an inter-species comparison of whole-body kinematics of walking and climbing insects (Theunissen et al., 2015).

In the *rod-climbing setup*, a horizontal rod was mounted above and perpendicular to a flat walkway (Figure 2, middle). The height of the rod varied between 5 and 50 mm above the walking surface, with heights of 18 mm or 36 mm used in the motion-capture experiments using the Vicon system. Animals were either video-recorded by a set of synchronized, orthogonally arranged, digital cameras (Basler 601af; this concerns Figure 6 only), by a single, top view, analog video camera and a slanted mirror next to the setup (Cohu; this concerns Figure 7 only), or motion-captured by the Vicon system mentioned earlier, as the animals touched the rod with their antennae and subsequently climbed it. A total of 262 motion capture trials from eight animals were included in the present analysis. As in searching trials, rod-climbing trials focused on the coordination of antennae and front legs. Accordingly, only joint position and joint angle time courses of both front legs and both antennae, along with the position time courses of the prothorax and head were recorded.

In the *searching setup*, only the flat walkway was used and animals were motion-captured as they approached the end of the walkway, stepped across the distal edge and engaged in bilateral searching movements of both front legs and both antennae (Figure 2, right), similar to the experiments described by Durr (2001). A total of 69 trials from three animals were included in the present analysis. In each trial, the motion capture analysis yielded the joint position and joint angle time courses of both front legs and both antennae, along with the position time courses of the prothorax and head. The same computational procedures were used as described by Theunissen and Dürr (2013).

In summary, the volume density estimates calculated for the limbs in the present study are based on 365 trials from 10 animals in case of hind and middle legs, 696 trials from 21 animals in case of the front legs, and 385 trials from 12 animals in case of the antennae.

### Body-Centered, Standardized Limb Coordinates

All volume density estimates were calculated using a standardized body shape on a 3D grid. In a first step, limb position coordinates were calculated separately for each limb and relative to the thorax- or head-fixed coordinate systems of the corresponding segment of the main body axis. From the original kinematic analysis (as described in detail by Theunissen and Dürr, 2013), each trial comprised absolute position coordinates of the limb segment boundaries (coxa, femur, and tibia of the legs, scape and pedicel/flagellum of the antennae), along with the six degrees of freedom of position and orientation of their carrying body segments, i.e., of the pro-, meso-, and metathorax for the front, middle, and hind legs, respectively, and of the head for the antennae. Whereas, the position of the body segment was used to calculate the relative position of the limb coordinates, the segment orientation gave the body-fixed, segment-specific coordinate system into which the corresponding relative limb coordinates were projected. The resulting, body-centered positions were scaled to the limb size of a standardized body shape, rounded to the nearest full millimeter, and counted on a 3D grid with 903 nodes, centered on the base of the limb (i.e., the thorax-coxa joints in case of the legs, and the head-scape joints in case of the antennae).

The standardized body shape was determined from the mean segment length and width measurements of the adult female specimens that contributed to the motion-capture data set. For each pair of limbs, a scaling factor B_ref_/B_curr_ was determined, where B_ref_ was the sum of standardized segment lengths of both femora, both tibiae and the carrying body segment in case of legs, and the sum of both standardized antenna lengths and the head length in case of the antennae. B_curr_ was the corresponding sum of segment lengths of the specimen that contributed the current trial. Thus, the scaling factor was adjusted for each pair of limbs, in order to account for variation of relative limb length among animals. The body-centered, standardized volume data grids of the eight limbs were then aligned in order to match the body segment lengths and location of the limb bases of the standard body shape. For this, the main body was assumed to be stiff and straight, neglecting movement of the thoracic joints and neck. The corresponding standardized body shape was used in all volume plots presented in this study in order to provide a 3D reference structure. Limb postures of this reference structure were set according to an arbitrary single instant of an experimental trial. The reference structure also includes the six tarsi. Since the motion-capture data did not comprise measurements of the tibia-tarsus angle, only the standardized tarsus length is drawn for reference. For the calculation of “action volumes” and “contact volumes” of the legs, the tarsi were assumed to be straight extensions of the tibia (see below).

### Tip, Contact, Action, and Affordance Volumes

One goal is to define an “affordance volume” that delimits a volume in which multiple limbs can act. In this volume, positions of one limb potentially provide an affordance for other limbs through an internal model. In order to find such an intersection volume, first the working ranges of the individual legs had to be charted. The physiological movement ranges of the eight limbs were calculated as density distributions across an orthogonal 3D grid of 1 mm spacing. Depending on the part of the limb considered, three types of volumes were calculated per limb: (1) the “action volume” comprised the movement range covered by the entire limb, i.e., the entire flagellum of an antenna, or the entire set of femur, tibia and tarsus of a leg. (2) The “contact volume” comprised the distal fraction of the flagellum in case of the antennae, or of the distal part of the tibia and entire tarsus in case of the legs. The default proximal limit of contact volume was 2/3 of the flagellum or tibia. The distal limits of contact volumes were determined separately for each leg, and ranged between 1.33 and 1.34 tibia lengths in front and hind legs, and between 1.38 and 1.39 tibia lengths in middle legs. These numbers correspond to the factor by which the tibia needed to be scaled in order to reach the tip of the tarsus. (3) Finally, the “tip volumes” were calculated from the movement ranges of the most distal points of the tracked limb segments, i.e., the antennal tips and tibia-tarsus joints of the legs.

**Figure 3:**
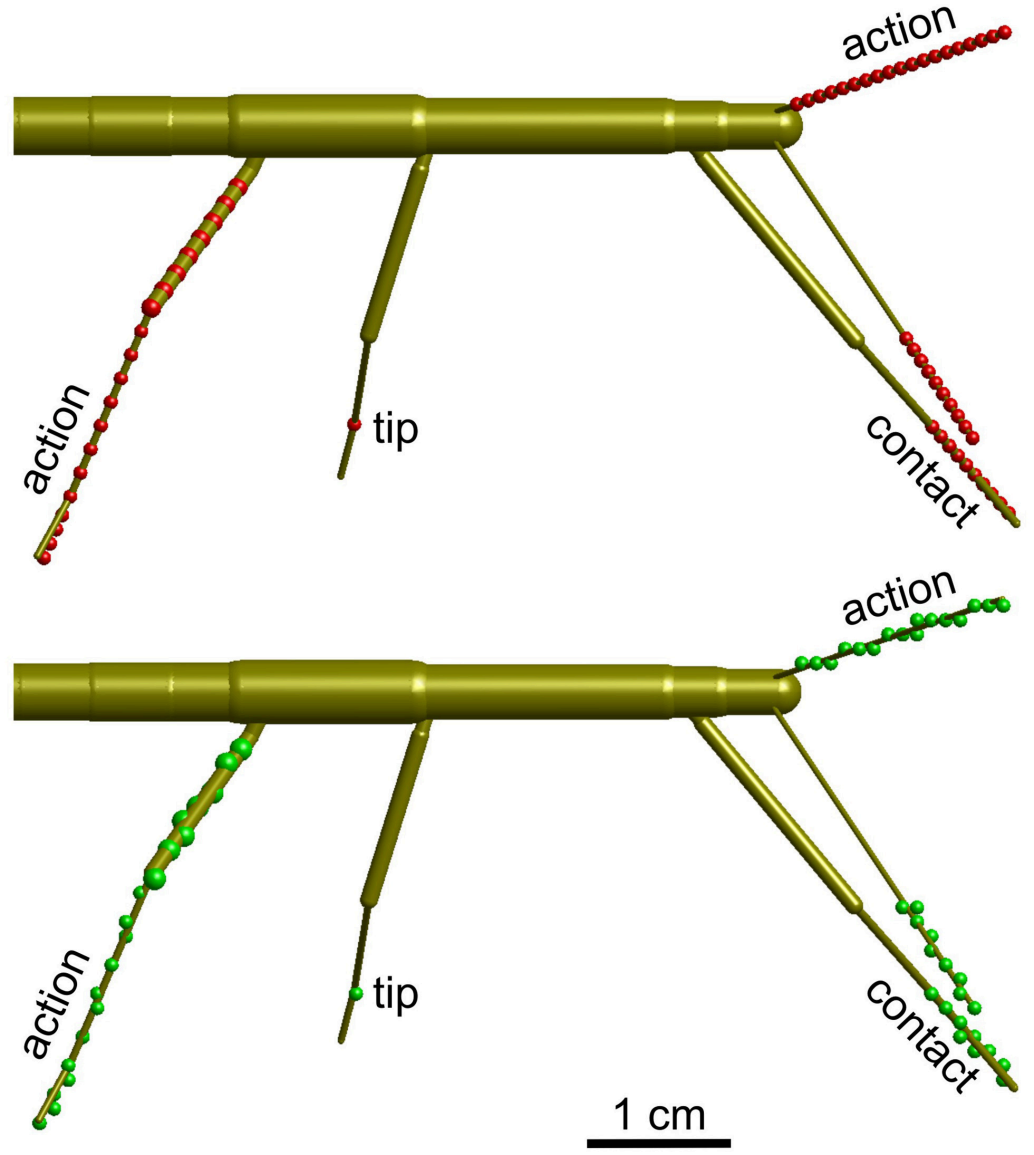
Calculation of volume density. Schematic top views of a stick insect show the points used for calculating volume densities (**top**, red dots), and the effect of mapping those points onto the 3D grid with 1 mm spacing (**bottom**, green dots). Three different types of volumes were calculated per limb. For the “action volume” of a leg, eight equidistant points along the femur and a further eight equidistant points along the tibia were determined per frame (red/green dots on the right hind leg). Additionally, four points were considered along the tarsus, which was assumed to be a straight extension of the tibia. In case of the antennae, the “action volume” was calculated from 20 equidistant points along the flagellum (dots on left antennae). For the “contact volumes,” 10 equidistant points were determined along the distal third of the tibia and the entire tarsus (see dots on right front leg). In case of the antennae, 10 equidistant points were considered for the distal third of the flagellum (see dots on right antenna). For the “tip volumes” only a single point per limb was considered. In case of a leg, this was the location of tibia-tarsus joint (single dot on right middle leg). In antennae, the distal end of flagellum was used (equivalent to the terminal dots on the depicted antennae).

In all cases, the volumes were calculated for a discrete set of points along the limbs. Figure 3 shows the distribution of these points for the three types of volumes calculated. In case of the contact volume, ten equidistant points were calculated along the tibia and tarsus as determined by a scaling factor. For antennae this scaling factor ranged between the proximal limit, i.e. 0.67, and the distal limit of 1.0. For legs, the scaling factor ranged between 0.67 and a distal limit between 1.33 and 1.39 (see above). Whereas, the flagellum can be considered reasonably straight (at least when it does not contact anything), the angle of the tibia-tarsus joint varies throughout a step with an approximate range between 90° (abducted) and 0° (aligned with the tibia). Since we had no information about the tibia-tarsus joint angle, we always assumed an angle of 0°, thus maximally extending the radial working range of the tibia. Given the difference in distance of the 10 points that were considered for each frame, increasingly distant points traveled increasingly longer arcs for a given excursion of the limb. To compensate for this effect, i.e., to avoid an overestimation of volume densities in proximal parts of the working ranges, each point was weighted with a factor. In case of n points (n = 10 for contact volumes), the weights were 2k/n/(n+1), with k = 1 …n. As a consequence, the sum of weights per frame was always 1. These volume densities provide a likelihood estimate for a limb to pass through that specific part of body-centered space, i.e. the grid.

Action volumes were calculated differently for antennae and legs. In antennae, the calculation followed the same principle as for the contact volume, except that the proximal limit was set to 0.1 and *n* = 20. In legs, eight equidistant points were distributed along the femur (also starting at a proximal limit of 0.1), a further eight along the tibia, and another four along the tarsus (Figure 3). Thus, 20 points per frame were used for the calculation of an action volume. In case of the antennae, these points were distributed equidistantly along the flagellum. The same weight distribution applied as explained above (*n* = 20) when updating the counts on the grid.

In order to estimate volume densities from absolute frequency distributions across the 3D grid, the count numbers per grid node were smoothed with a cubic kernel of spanning 53 grid nodes. This kernel had a Gaussian weight distribution with standard deviation of 1 and a sum of weights equal to 1. To obtain reasonably smooth volume boundaries, we chose a volume density threshold that was equivalent to 1% of the maximum density per limb and volume type. This threshold limited the volume to a range of 95.3 to 98.6% of the summed density values, depending on the type of volume and limb. The detailed values are listed in Supplementary Table 1.

Finally, affordance volumes were calculated as the intersecting volume of two neighboring limbs, e.g., the right middle and hind legs, or the left antenna and front leg. All calculations and volume visualization were done in Matlab R2018a (The Mathworks, Natick/MA), including the Geom3D toolbox of David Legland. Transparent volume surfaces were calculated by use of the Matlab function *boundary()*, using a convexity scaling factor of 0.8, with 1.0 being no convexity between the supporting polygon nodes.

### Artificial Neural Network Simulations

Pairs of non-spiking Artificial Neural Networks (ANN) were used to learn mappings between joint angle spaces of neighboring legs. A foot position in space that can be reached by two neighboring legs corresponds to a set of joint angles for each one of these legs. We used neural networks of passive summation elements to transform the joint angles of one leg to the corresponding set of joint angles of the neighboring leg for identical foot positions in space. The training data were obtained from the grid points contained by the affordance volumes spanned by any one of the four ipsilateral pairs of legs. For each point within an affordance volume, the corresponding sets of joint angles were calculated for both legs, using the inverse kinematics calculation as deduced by Cruse and Bartling (1995). Accordingly, we assumed fixed and slanted rotation axes for the thorax-coxa joints, such that protraction /retraction about the thorax-coxa joint correlated with pronation/supination of the leg plane. This simplification is justified also in freely walking and climbing stick insects, as protraction/retraction and pronation/supination angles are strongly correlated in these conditions (see, Figure 11 of Theunissen et al., 2015). The corresponding Euler angles of the ThCx joint axis are given as yaw and pitch angles of the resting coxa in Table 1.

**Table 1:** Standardized body shape: segment lengths, insertion coordinates, roll and pitch angles of the coxae as used for inverse kinematics.

**Limb**	**Carrying segment, length [mm]**	**Insertion coordinates, x, y, z [mm]**	**Yaw, pitch [deg.]**	**Total length [mm]**	**Coxa, length [mm]**	**Femur, length [mm]**	**Tibia, length [mm]**	**Tarsus length [mm]**
Left antenna	Head: 4.30	3.88, 0.95, 0.84	–	34.42	–	–	–	–
Right antenna	Head: 4.30	3.87, −0.95, 0.84	–	34.44	–	–	–	–
L1	Prothorax: 3.76	2.03, 1.33, −0.73	84, 34	38.67	1.32	16.57	15.13	5.24
R1	Prothorax: 3.76	2.03, −1.33, −0.73	−84, 34	38.60	1.32	16.48	15.61	5.18
L2	Mesothorax: 17.45	1.03, 1.70, −1.11	92, 37	29.68	1.42	12.12	11.64	4.51
R2	Mesothorax: 17.45	1.03, −1.70, −1.11	−92, 37	29.76	1.42	12.16	11.75	4.44
L3	Metathorax: 11.87	1.34, 1.67, −1.05	114, 29	35.64	1.54	14.55	14.65	4.77
R3	Metathorax: 11.87	1.34, −1.67, −1.05	−114, 29°	35.51	1.54	15.60	14.71	4.79

As a result, each point within an affordance volume yielded 2x3 joint angles, i.e., protraction, levation and extension angles of two neighboring legs (e.g., the right front leg and the right middle leg). The ANNs were trained to map three of these angles, i.e., the posture of a “sender leg,” to the other three angles, i.e., the posture of a neighboring “receiver leg.” The input of such a feed-forward ANN can be considered the posture of the sender leg, the output can be considered the corresponding target posture of the “receiver leg.” An affordance is thus generated in the following way: if the receiver leg was moved so as to assume this target posture, the position of its tibia-tarsus joint would coincide with that of the “sender leg.” Two reciprocal mappings were learned for each affordance volume. Each one of the two legs was once used as the sender leg (joint angles were used as an input to the ANN) and once as the receiver leg (joint angles were used as training values for supervised learning of the appropriate output of the ANN).

Based on the experimental data, the affordance volumes of the left front and middle legs comprised 5,500 matching pairs of leg postures (8,382 on the right side). In case of the left middle and hind legs, the affordance volume comprised 2,918 matching pairs of leg postures (3,741 on the right side). For training and evaluation of each ANN, the corresponding data set was split into a training part (80%, 4,400 samples for the left side and 6,705 for the right side) and a testing part (the remaining 20%). The testing part of the data set was used to evaluate the generalization capabilities of a trained ANN, assessing how well it could interpolate for data points it had never encountered during training.

Feed-forward ANNs were used with systematic changes of the network complexity. As a baseline, a feed-forward NN without a hidden layer was used. Since this network structure is equivalent to a regression problem, an optimal solution was found analytically using the normal equation and through calculation of the pseudo-inverse. In all other cases, the ANNs contained a single hidden layer. The size of this single hidden layer was changed systematically in order to assess mapping performance for different network complexities. The Keras framework (https://keras.io/) was used for ANN training, with sigmoid activation functions in hidden layer neurons and linear activation functions in the output layer neurons. Networks were trained in batches of ten, using the optimizer ADAM (Kingma and Ba, 2015). ADAM implements an adaptive gradient descent method that includes a momentum term and has the advantage that it does not require any additional hyperparameters. Weight matrices were initialized at random, using the Glorot uniform initialization (Glorot and Bengio, 2010). Training was repeated in five individual runs for the data of the left legs. ANNs for the right leg pairs were trained only once for comparison. Training runs lasted for 5000 epochs, which proved to be sufficient for convergence. Sample data and ANN training code are publicly available under (https://pub.uni-bielefeld.de/record/2932236) (Schilling and Dürr, 2018).

## Results

Based on our considerations about peripersonal space as the volume within which the body and its limbs may physically interact with the environment, we first calculated the action volumes of all legs and antennae. The combination of these action volumes then delineated the boundary of what we propose to call the peripersonal space of an insect. In contrast, the intersection of each pair of action volumes was equivalent to the joint working range of two neighboring limbs. This was termed the affordance volume of a pair of limbs.

### The Combined Action Volumes of all Limbs Delineates Peripersonal Space

**Figure 4:**
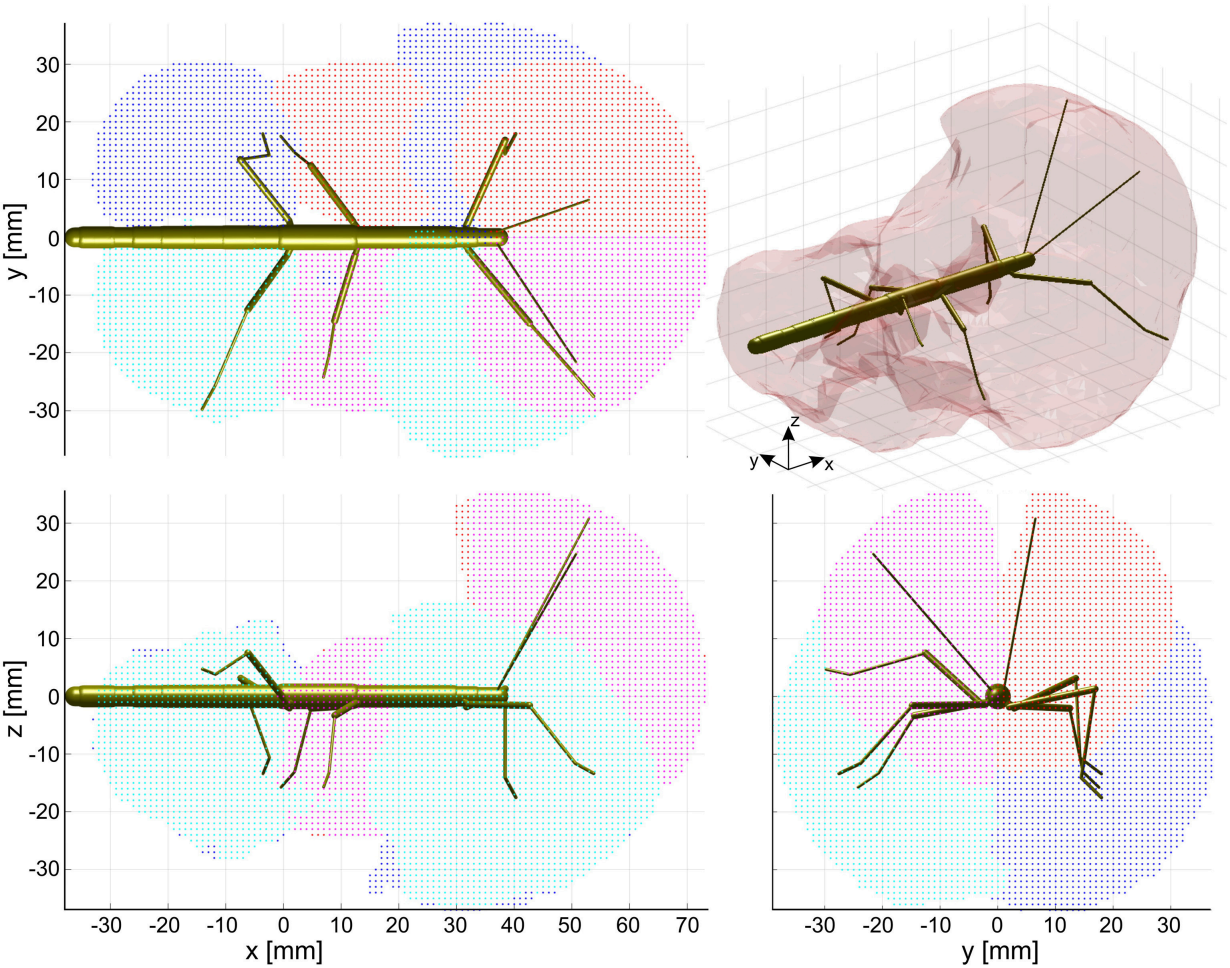
Action volumes of the eight limbs. Orthogonal projections of the action volumes of all six legs and two antennae, depicted as colored points on the 1 mm grid that was used to calculate volume densities. Red and dark blue dots show the volumes of left limbs, magenta and light blue dots those of right limbs. Red/magenta show volumes of antennae and middle legs, dark/light blue dots show volumes of front and hind legs. Top, side and frontal views (as indicated by the standardized insect in the background) are aligned and scaled to match. Top right: The combined action volume of all limbs, delimited by a transparent envelope surrounding the non-zero grid points shown in the orthogonal projections. Note that volumes for left and right limbs were calculated separately. As a result, they are similar but not the same.

The action volume of a limb was defined as that part of space, where this particular limb could contact an external object, irrespective of which part of the limb was making contact. Action volumes were calculated from a large motion capture data set, comprising a total of 6061.5 s (1 h 41 min) of movement sequences from 365 to 696 experimental trials (depending on the kind of limb, see Material and Methods) of the Indian stick insect *Carausius morosus*. The experimental data had been acquired in three different locomotion experiments, including climbing and searching episodes (Figure 2). Two hundred single limb postures per second were sampled, so that even fast limb movements were broken down into a reasonable set of discrete postures. For example, a typical swing movement of a leg was represented by some 40 limb postures. For simplification, the movements of the neck and of the two thorax joints were neglected, so that the insertion points of the limbs were fixed before calculating the body-centered coordinates of each limb segment. Furthermore, the volumes of the limbs themselves were neglected and each limb posture was treated as a set of 20 points on a 1 mm grid. As a consequence, the antennal posture was treated as a set of points along a single line, and each leg posture was treated as a set of points on a pair of lines: one line for the femur and another line for the tibia and tarsus (Figure 3). To estimate the shape of an action volume, we first approximated the likelihood of the limb to pass through a particular point in body-centered space, and then set a density threshold to determine the volume boundary (for details on the likelihood approximation, in particular the spatial smoothing procedure and the compensation of decreasing likelihood with increasing distance from the insertion point, see section Tip, Contact, Action, and Affordance Volumes). As a consequence, the actual shape of the action volume strongly depended on the particular choice of density threshold. In all figures shown in this study, we applied limb-specific thresholds equivalent to 1% of the maximum density recorded for a particular limb. Supplementary Table 1 lists the limb-specific threshold values and the corresponding fraction of the total volume density comprised by the action volume (which was always > 95%). The combined action volumes of all eight limbs are shown in Figure 4. The orthogonal projections of the grid points reveal that the action volumes of the left and right limbs of the same segment have similar shapes, though not the same. Throughout this study, we did not pool data for limbs of the same segment.

The action volumes of the front legs were the largest of all limbs, amounting to more than 60 ccm. This was approximately twice the action volume of the antennae and approximately three times that of the middle and hind legs (Table 2). The action volumes of the middle legs were the smallest of all limbs, amounting to 88 and 93% of the hind leg action volumes in left and right legs, respectively. The order of action volume size was the same as the order of limb length, with the front legs being the longest and the middle legs being the shortest (Table 1). However, the ratio of front leg length over antenna length was only 112%, which is substantially smaller than the corresponding volume ratio of about 200%. Similarly, the ratio of front leg length over middle leg length was 130%, compared to about 300% for the volume ratio. We conclude that the front legs were the most agile limbs and covered much larger ranges than any other limb. Since the leg length ratios of middle and hind legs (83 and 84% for left and right legs, respectively) were smaller than the corresponding volume ratios, middle legs proved to be more agile than hind legs.

**Table 2:** Comparison of the derived limb volumes (using the 99% threshold for the likelihood as explained in section Tip, Contact, Action, and Affordance Volumes) of the four limb pairs.

	**Hind leg**	**Middle leg**	**Front leg**	**Antenna**
**LEFT**				
Tip	13.023; 60%	13.626; 68%	46.330; 76%	12.082; 38%
Contact	19.882; 92%	20.677; 104%	65.630; 108%	27.227; 86%
Action	21.542; 100%	19.937; 100%	60.786; 100%	31.615; 100%
**RIGHT**				
Tip	14.884; 64%	13.589; 66%	39.436; 64%	10.751; 35%
Contact	23.112; 99%	21.757; 106%	61.113; 100%	24.766; 81%
Action	23.290; 100%	20.484; 100%	61.345; 100%	30.531; 100%

*Rows indicate volumes in ccm and fraction of the action volume for tip volume (top), contact volume (middle), and action volume (bottom)*.

**Figure 5:**
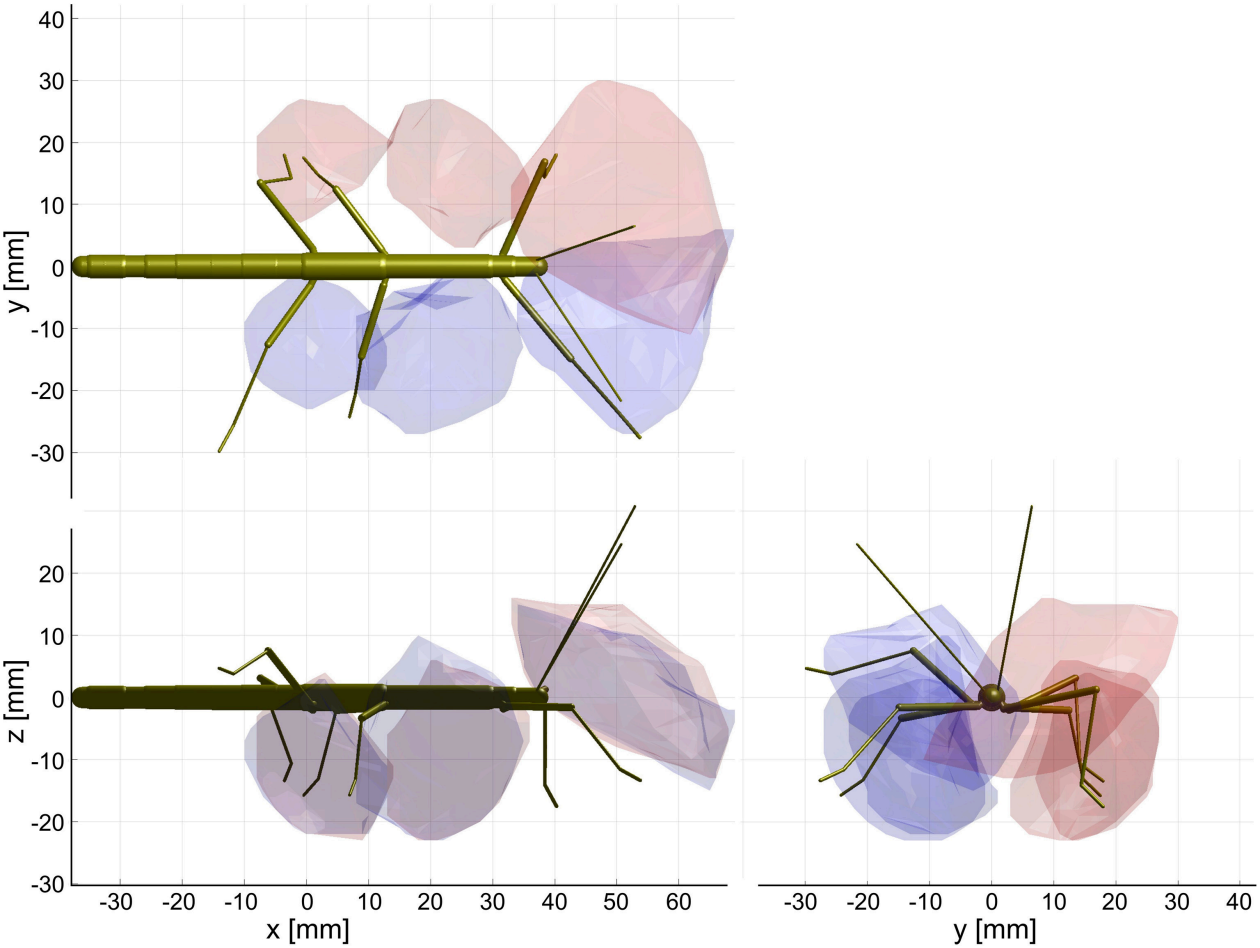
Affordance volumes for ipsilateral limb pairs. Orthogonal views of the three left (*red*) and right (*blue*) affordance volumes, delimited by transparent envelopes. Each affordance volume corresponds to the overlap of the action volumes of an ipsilateral limb pair (*front*: antenna × front leg; middle: front leg × middle leg; *rear*: middle leg × hind leg). Top, side and frontal views are indicated by the standardized insect in the background. All views are aligned and scaled to match. Note that volumes for left and right limbs were calculated separately. As a result, they are similar but not the same.

The shape of the combined action volumes of all limbs reveals a nearly hemispheric region of about 35 mm radius around the head (spanned by antennae and front legs), and a dorso-ventrally compressed volume ranging from mid-mesothorax rearward along the first three quarters of the abdomen. Note that Figure 4 conceals the overlap of neighboring action volumes. These overlap volumes proved to cover substantial fractions of the action volumes (Figure 5). For example, the overlap between antennal and front leg action volumes amounted to 14 and 10% of the action volumes of left and right front leg, respectively (for volume sizes in ccm, see Table 3). This means that 10–14% of possible contact locations of a front leg may be contacted also by the ipsilateral antenna. In other words, bidirectional transfer of spatial information from one limb to another is possible in these overlap volumes, thus potentially giving rise to affordances. Accordingly, we chose to call these overlap volumes *affordance volumes*. The affordance volumes of front and middle legs shown in Figure 5 covered 32 and 45% of the left and right middle leg action volumes, respectively. The affordance volumes of middle and hind legs corresponded to 20 and 24% of the left and right hind leg action volumes. The lower left side view in Figure 5 reveals that the affordance volumes of ipsilateral leg pairs are located mostly below the body axis. This is not the case for the affordance volumes of antennae and front legs which appear almost centered on the horizontal plane through the body axis. Note that the top and frontal views in Figure 5 reveal a zone of bilateral overlap between the left (red) and right (blue) affordance volumes of antennae and front legs. This narrow, elongate region in front of the insect head indicates that both antennae and both front legs could transfer contact information among each other. This region comprises the volume that is covered by the outstretched front legs aligned with both antennae, as it occurs in the posture that *Carausius morosus* assumes for its camouflaging twig mimesis.

**Table 3:** Affordance volumes for ipsilateral limb pairs.

	**Leg3/Leg2**	**Leg2/Leg1**	**Leg1/Ant**
**LEFT**			
Tip	2.635; 62%	4.233; 66%	0; 0%
Contact	4.864; 114%	7.825; 122%	6.702; 77%
Action	4.263; 100%	6.423; 100%	8.736; 100%
**RIGHT**			
Tip	3.279; 65%	5.530; 61%	0; 0%
Contact	6.072; 120%	10.551; 116%	2.985; 50%
Action	5.080; 100%	9.135; 100%	5.963; 100%

*Volumes were calculated as overlap of the corresponding tip (top row), contact (middle row), or action volumes (bottom row) in ccm. All volumes are indicated as a fraction of the corresponding affordance volume based on action volumes*.

### Behavioral Relevance of Contact Location

**Figure 6:**
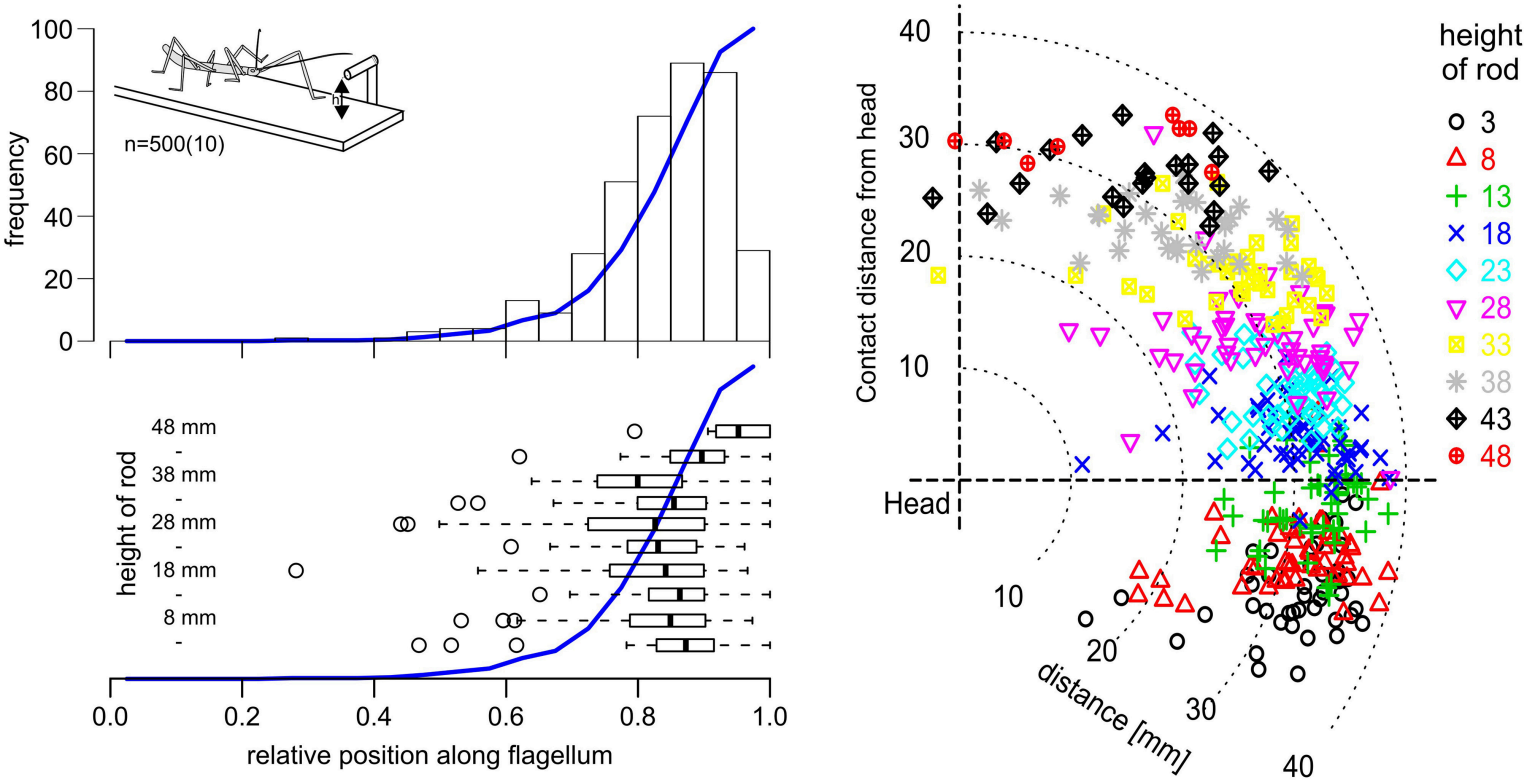
Initial antennal contacts occur in the distal third of the antennal action volume. **Top left**: Location of the first antennal contact along the flagellum, as a stick insect walks toward a horizontal rod that is reaching across the walkway at height h (see insert). Histogram of five trials per 10 rod heights per 10 animals. Blue line shows the cumulative sum. Most initial contacts with an obstacle of this kind occur along the distal third of the flagellum. **Bottom left**: Box-whisker plots show medians, IQR and min/max ranges of distributions of contact locations, separately for each rod height (*n* = 50, each). Open circles show outliers. Except for the highest obstacle heights, medians and IQR are very similar. **Right**: Contact locations in head-centered coordinates (side view), with different colors corresponding to different rod heights. Most initial contacts were located in the distal third of the action volume of the antenna, here approximately between 26 and 39 mm away from the antennal base.

Affordance volumes, as defined here, comprise positions suitable for coordinate transfer among ipsilateral limbs. This leads to the question whether these volumes were not just computationally plausible but also behaviourally relevant. After all, the affordance volumes shown in Figure 5 had been calculated based on the action volumes of entire limbs, including parts of the limb which would be at least awkward, if not unlikely contact locations in natural behavior. For example, whereas it is trivial to observe that an insect regularly contacts obstacles with one if its feet, this is not clear at all for more proximal parts of the limb, such as the femur. To address this question, we observed stick insects as they climbed a horizontal rod that was held across the walkway, and recorded the contact locations along the antennae and front legs. In order to have independent position records from contact to contact, only the location of the initial limb contact was recorded per trial. Figure 6 shows the result for the antennae, including 500 single trials from 10 animals and 10 different rod heights. The results clearly show that initial antennal contacts with a horizontal rod occur almost exclusively in the distal half, and approximately 90% occur in the distal third of the antennal flagellum (Figure 6, top left). This is largely independent of the height of the rod (Figure 6, lower left), as the median relative contact location along the flagellum ranged between 0.8 and 0.9 in almost all cases, and shifted distally only for very high rods (43 mm and above). Accordingly, most initial contact locations were at least 20 mm away from the head, irrespective of whether the rod was located above or below the body axis (Figure 6, right).

**Figure 7:**
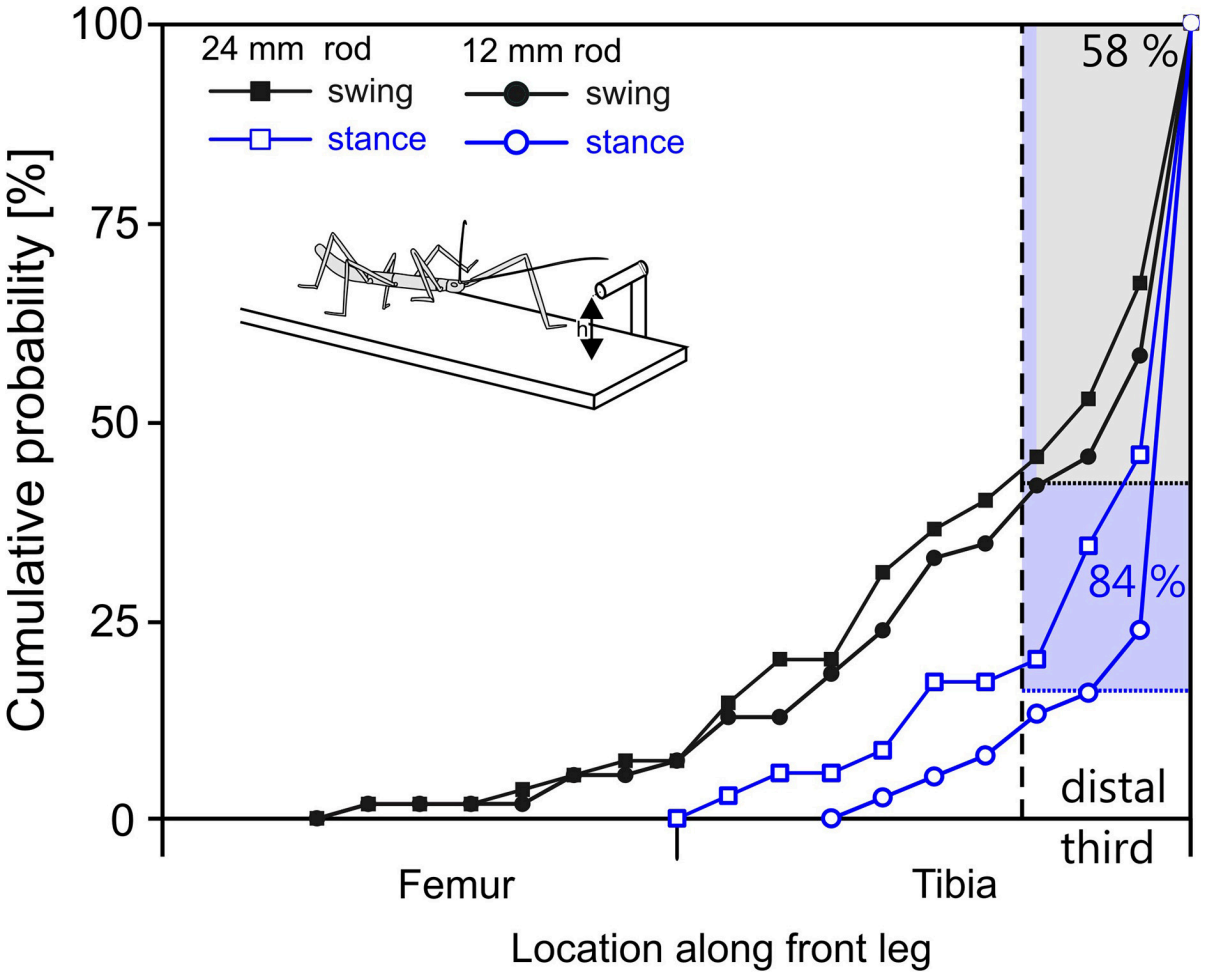
In reaching movements, the front leg contacts a horizontal rod most often with the distal tibia or tarsus. Cumulative probability plots of initial leg contact location along the length of the leg (location shown on x-axis is standardized to 50% femur + 50% tibia; contacts by the tarsus are counted as 100% leg length). Trials were separated according to rod height (squares: 12 mm, circles: 24 mm) and depending on whether the initial antennal contact occurred during swing (black) or stance (blue) of the subsequently reaching front leg. The targeting quality of reaching movements initiated during stance is superior to those initiated during swing. 84% of initial contacts occurred in the distal third of the tibia or on the tarsus when reaching followed a first contact during stance movement (blue). When reaching required re-targeting of an ongoing swing movement (black), 58% of first contacts occurred in the distal third of the tibia or at the tarsus.

The situation was more variable in case of leg contacts. Stick insects are known to respond to antennal contact with altered swing movements of the front legs (Schütz and Dürr, 2011). Two kinds of responses can be distinguished, depending on the state of the front leg at the time of contact by the ipsilateral antenna. If the front leg is in stance phase in the instant of antennal contact, the front leg completes the stance movement and then lifts off to execute a reaching movement that often is considerably higher than normal. If the front leg is in swing phase in the instant of antennal contact, the front leg often executes a re-targeting movement with a distinct upward kink in the trajectory. In the latter cases, the leg can be very close to the object as the antenna makes contact, leaving little reaction time before hitting the object with a part of the leg. Accordingly, our results showed that the distribution of initial contacts along a front leg depended in large parts on whether antennal contact had been made during swing or stance (Figure 7, compare black with blue lines). In comparison, the effect of rod height was small.

As can be seen in Figure 7, the probability of initial contact on the femur was very low in case of stance-initiated movements, and zero for swing-initiated movements. Contact probability was highest in the distal third of the tibia and on the tarsus. Initial contacts were recorded in this region in 58% of trials with swing-initiated movements, and in 84% of trials with stance-initiated movements. Following leg contacts with the tarsus, the animal typically grasped hold of the rod. When the rod was contacted with the distal tibia, the leg was typically retracted until the tarsus achieved firm grip. For other contact locations, the leg was lifted and retracted until another contact was achieved.

### Limb Contacts and Affordance Space

**Figure 8:**
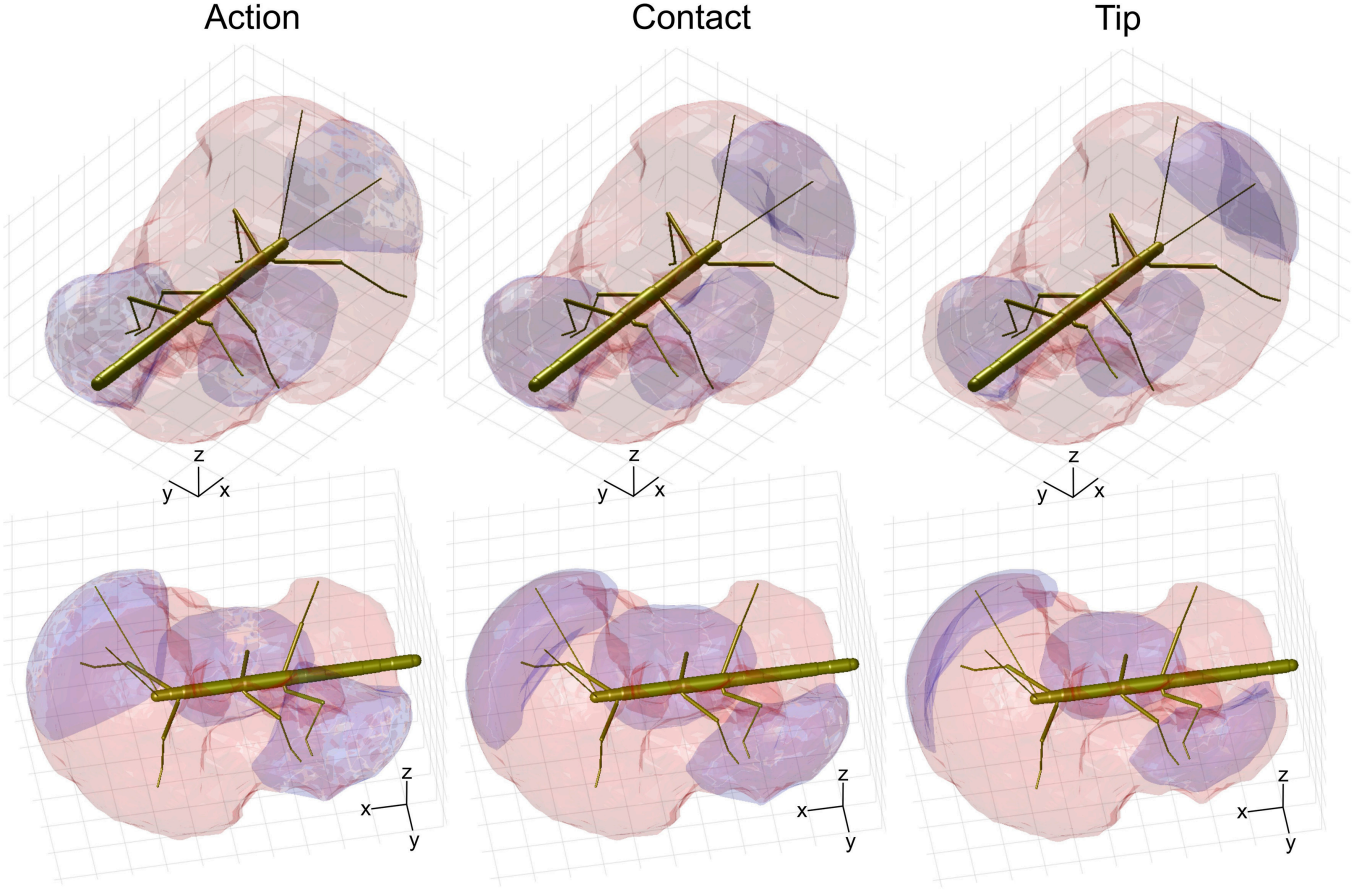
Comparison of action, contact and tip volumes. **Left**: Action volumes of the right antenna, right middle leg and left hind leg are shown as blue regions within the envelope of the peripersonal space (red). **Middle**: Contact volumes. **Right**: Tip volumes. Each column shows two views of the same figure. The coordinate system inserts show 10 mm scale bars along the longitudinal (x), transverse (y), and vertical (z) axes.

Given the results shown in Figures 6, 7, we wanted to know how the shapes of the action volumes would change if only those parts of a limb were considered that were likely to contact an obstacle. To test this, we calculated the “*contact volumes*” for all limbs. The computational procedure was the same as for the calculation of action volumes, except that only 10 points per limb posture and frame were considered for volume density estimates. These 10 points were placed along the distal third of the antenna or along the distal third of the tibia and the entire tarsus. For immediate comparison of action and contact volumes, Figure 8 shows the volume envelopes of the right antenna, right middle leg and left hind leg within the peripersonal space. In case of the antenna, the neglect of the proximal two thirds resulted in a fairly wide gap between the head and the contact volume. As a consequence, the antennal contact volumes comprised only 82 or 86 % of the corresponding action volumes in left and right limbs, respectively (Table 2). For comparison, we also calculated the volumes for the most extreme reduction of contact sites on a limb, i.e. a single point. Such *tip volumes* (Figure 8, right column) were calculated from the volume densities of the most distal point of the motion-captured limb segment (the tip of an antenna or the tibia-tarsus joint of a leg, see Figure 3). As expected, the tip volumes of the antennae were very narrow curved, convex regions (see Figure 8, lower right). Despite their small width, antennal tip volumes still comprised 38 and 35% of the left and right antennal action volumes, respectively.

Compared to the relatively strong size reduction of antennal contact volumes, the contact volumes of the legs were of nearly the same size and shape as their corresponding action volumes (Figure 8 and Table 2). In fact, three of the six contact volumes turned out to be even slightly larger. We attribute this apparent increase in volume to slight weighting differences of the discretised limb postures for the calculation of the volume densities for action and contact volumes. These differences lead to different threshold values and, as a consequence, in variation of volume size and shape. Comparing the action and contact volumes of the hind and middle legs in Figure 8 reveals that the gap between the contact volumes and the body is relatively small. This can be explained by strongly flexed leg postures that let the distal tibia and tarsus come very close to the base of the leg. As a consequence, much of the volume that is traversed by the femur may also be traversed by the foot and distal tibia. The most pronounced difference between action and contact volumes of the legs appears to be the region traversed by the “knees” (femur-tibia joint) and the nearby distal femur and proximal tibia. A foot could only reach knee positions of postures with moderate levation of the femur. This is because the foot can move to the previous knee position only by a combination of strong levation of the coxa-trochanter joint and strong flexion of the femur-tibia joint.

The strong effect of flexed leg postures becomes evident when comparing the tip volumes of the legs (Figure 8, right column) with their corresponding action volumes. Other than the antennal tip, that cannot be moved close to the head, the tibia-tarsus joint can be moved very close to the base of the leg, allowing this joint to traverse a substantial fraction of the action volume of the entire leg. Accordingly, Table 2 lists the ratios of tip volume over action volume of the legs as ranging between 60 and 76%, which is approximately twice the ratio for an antenna (35–38%).

**Figure 9:**
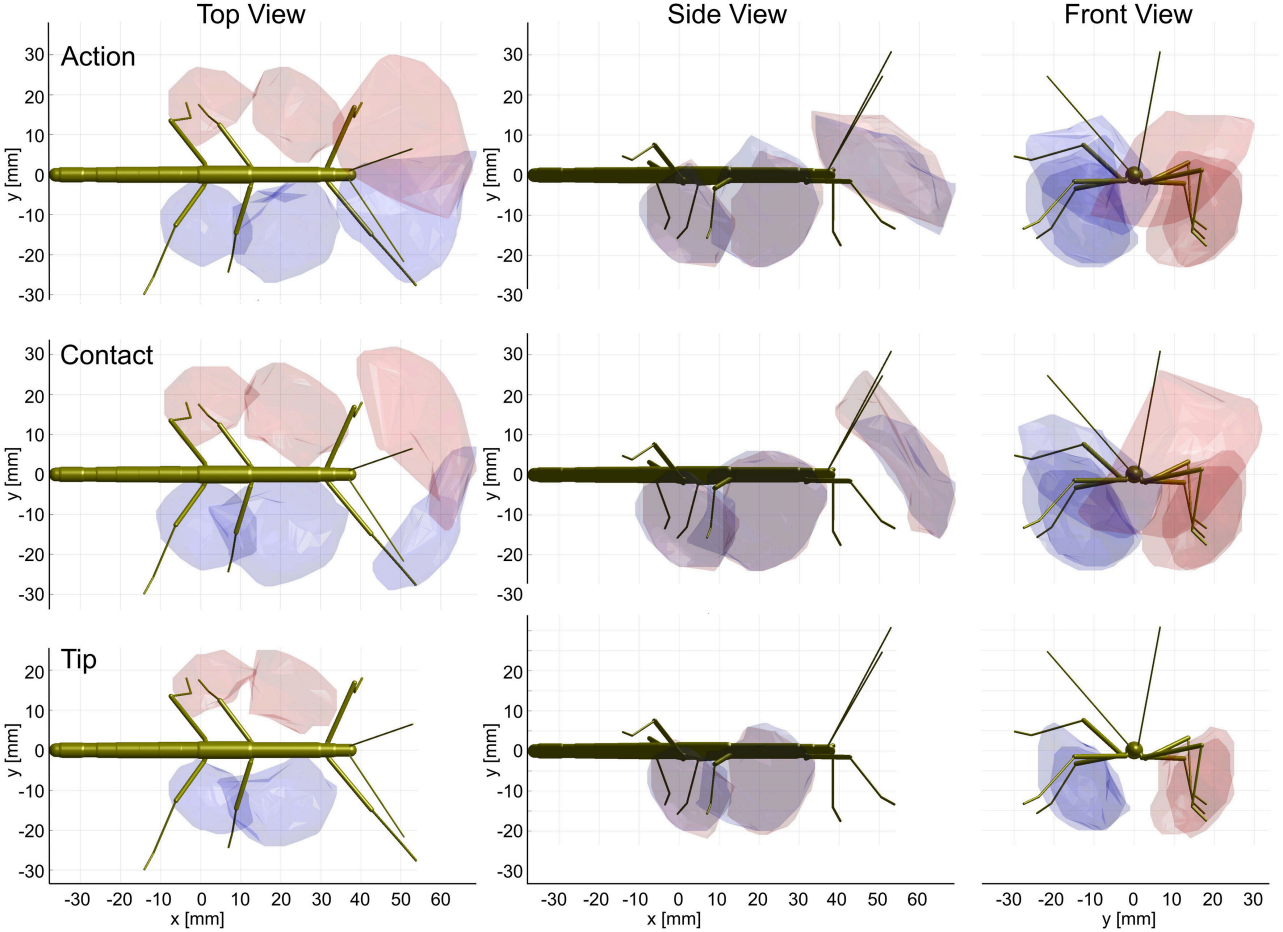
Action and contact volumes give rise to similar affordance volumes. Panel rows show the three volume types used for calculation of the corresponding affordance volume. Panel columns compare the top, side and front views of different affordance volumes, respectively. Left leg (*red*) and right (*blue*) affordance volumes are shown as transparent envelopes, equivalent to the overlap of the corresponding pairs of action, contact or tip volumes (*front*: antenna × front leg; Middle: front leg × middle leg; *rear*: middle leg × hind leg). All views are aligned and scaled to match. Note that, if the tip volumes were considered, there were no affordance volumes for antennae and front legs because the tibia-tarsus joint never reached as far as the tip of the ipsilateral antennal tip.

Having established similar properties for action and contact volumes, we reasoned that the overlap of contact volumes for ipsilateral pairs of legs should not differ much from the overlap of action volumes. In other words, the affordance volume for a given pair of legs should remain the same even if the underlying volume density estimates were calculated from a subset of points per limb posture. Indeed, this was the case. Figure 9 juxtaposes affordance volumes based on action, contact and tip volumes, revealing strong similarity between all leg affordance volumes, particularly of those based on action and contact volumes. The absolute sizes of affordance volumes and their relative size compared to the corresponding action volumes are listed in Table 3. The data show that the affordance volumes of antennae and front legs were affected much more strongly by the restriction to contact regions than the affordance volumes of leg pairs. This is because the antenna maintains a fairly straight posture during movement, such that the distal part of the antenna can only be reached by relatively strong extension of the front leg. As a consequence, that part of the front leg contact volume that required a flexed leg posture was excluded from the affordance volume, despite the fact that the contact volume of a front leg changed only little compared to its action volume. For the same reason there is no overlap of antennal and front leg tip volumes at all. The tibia-tarsus joint of a front leg cannot reach the tip of the ipsilateral antenna.

Much like it was observed for the comparison of contact and action volumes in Table 2, affordance volumes based on contact volumes proved to be even larger than those based on action volumes (between 114 and 122%). However, as outlined in conjunction with Table 2, differences in weighting entail relatively small differences of the volume density threshold used to delimit the boundary, causing a variation of volume size. Since affordance volumes are considerably smaller than contact volumes, the relative variation in size was larger for the affordance volumes (Table 3) than for the contact volumes (Table 2).

In summary of the experimental results, we propose to distinguish two kinds of spatial regions surrounding the insect body that differ in their behavioral relevance. The first of these is what we called peripersonal space. In analogy of the use of that term in human psychology and neuroscience, it comprises that part of the ambient space that is “within reach” of any body parts, the limbs in particular. In the present study we defined it as the combination of all action volumes of the limbs, as shown in Figure 4, top right. The second region is what we propose to called *affordance space* and defined as the intersection of action volumes of all limb pairs. The functional significance of this distinction is that the affordance space is “within reach” of at least two limbs and therefore allows a coordinate transfer that is suitable for the control of aimed limb movements based on a physical contact of another limb. Based on our considerations about behavioral relevance, we suggest that affordance volumes should be related to those regions, where spatial contacts are likely to occur in natural behavior.

### Modeling Coordinate Transfer Within the Affordance Space

Given the definition of affordance space above, we wanted to know how complex a computational mapping would have to be that mediates coordinate transfer within the experimentally derived affordance volumes as shown in Figure 5. To this end, we studied the computational properties of the transformation of postures between neighboring legs (in both directions: backwards, from an anterior leg to a posterior leg, and forwards, i.e., in the opposite direction). We used two different methods, both related to feed-forward Artificial Neural Network (ANN) simulations, but of different complexity. For an immediate mapping of a set of three joint angles (the posture of the sender leg) to another set of three joint angles (the target posture of the receiver leg), we calculated an optimal linear regression. This then served as a benchmark for comparison with more complex ANN structures that included a hidden layer of variable size. Our goal was to determine how the accuracy of the posture mapping depends on the complexity of the underlying neuronal network structure.

For the two affordance volumes of left leg pairs (front-to-middle-leg, middle-to-hind-leg) a simple regression provided only a coarse approximation of the target values (Table 4): for the front-to-middle-leg transformation the mean squared error (MSE) was 61.0, equivalent to a mean error of around 7.8° per leg joint. The middle-to-hind-leg transformation achieved a smaller MSE of 10.0, equivalent to around 3.2° per leg joint. This difference in mapping accuracy can be explained by the larger size of the affordance volume of front and middle legs, making the approximation of joint angle transformations by a simple hyperplane more error-prone. Table 4 lists the joint angle working ranges and the MSE for each degree of freedom. For the transformation in the opposite direction, i.e., from a posterior sender leg to an anterior receiver leg, the MSE dropped for the middle-to-front leg pairing to 34.3 (average of 5.9° per leg joint) and rose for the hind-to-middle leg pairing to 18.2 (average of 4.3° per leg joint).

**Table 4:** Joint angle range inside the affordance volume.

**Degree of freedom**	**Protraction/retraction**	**Levation/depression**	**Extension/flexion**
**AFFORDANCE VOLUME: LEFT FRONT AND MIDDLE LEG**			
Front leg [min, max]	−22.9°, …, 72.5°	−114.1°, …, −1.3°	42.9°, …, 158.9°
Middle leg	−76.6°, …, −1.4°	−114.6°, …, 26.4°	0.3°, …, 136.3°
**REGRESSION:**			
Optimal front-to-back projection, MSE	34.5	45.3	103.1
Optimal back-to-front projection, MSE	48.3	32.3	22.4
**AFFORDANCE VOLUME: LEFT MIDDLE AND HIND LEG**			
Middle leg [min, max]	−4.6°, …, 62.4°	−95.6°, …, 22.5°	2.0°, …, 113.1°
Hind leg	−52.8°, …, 10.6°	−120.7°, …, 7.8°	59.2°, …, 153.8°
**REGRESSION:**			
Optimal front-to-back projection, MSE	13.7	8.4	7.9
Optimal back-to-front projection, MSE	7.1	13.8	33.8

Overall, these results show that a regression yields a poor approximation of a joint angle mapping. The variability of the different mappings further stresses the high non-linearity of the space. Therefore, we employed more complex models, including a hidden layer of varying size.

### Comparison of Different Model Complexities

**Figure 10:**
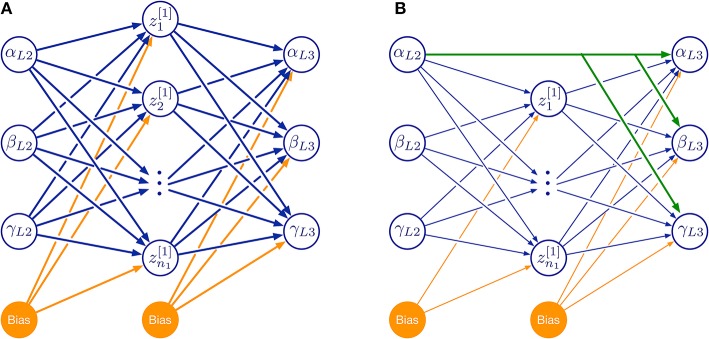
Feed-forward neural network topologies used in this study. **(A)** Three-layered network with hidden layer of variable size. **(B)** As in **(A)**, but with additional skip connections that shortcut the hidden layer. Both examples map three joint angles (α, β, γ) of the left middle leg (L2) to a corresponding set of three joint angles of the left hind leg (L3) via a hidden layer (neurons labeled by z and subscript numbers from 1 to n1). Information flow is from left to right:. Additional bias neurons are shown in orange. The size of the hidden layer was altered systematically by increasing n1. Green connections in **(B)** show an example of skip connections that directly connects an input neuron to all output neurons.

For a systematic investigation of the required model complexity, we trained ANNs with two kinds of architectures (Figure 10). The first of these architectures was a three-layered feed-forward ANN with varying number of hidden neurons (Figure 10A). The second architecture additionally included skip connections that shortcut the hidden layer (Figure 10B). As before, all simulations were done for the two left affordance volumes of leg pairs.

**Figure 11:**
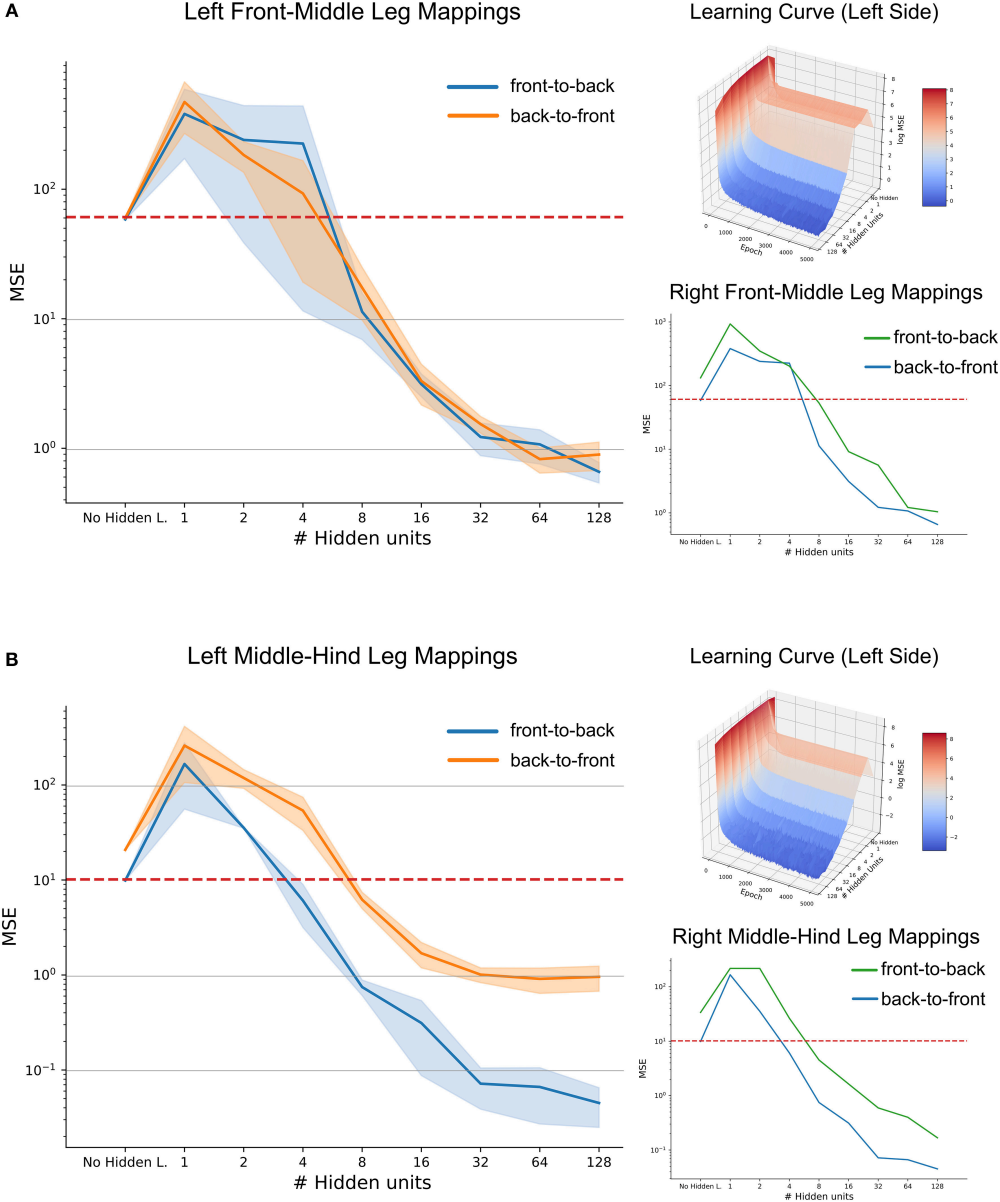
High-accuracy mappings were obtained for reasonably small networks. Average mean-squared error at the end of five training runs as a function of hidden layer size. **(A)** co-ordinate transfer between left front and middle legs. **(B)** As in **(A)** but for left middle and hind leg. Large left panels compare mapping accuracy for front-to-back (blue) and for the reciprocal back-to-front projection (orange). Top right inserts show accuracy as a function of both hidden layer size and training duration, illustrating how the test error improved over time. Lower right inserts compare accuracy-complexity functions of the right pair of legs (green, front-to-back projection) with that of the left pair of legs (blue).

When evaluated on a set of previously unseen test data, the mean performance of the three-layered ANNs as a function of hidden layer size is shown in Figure 11, along with the benchmark accuracy achieved by regression. The blue shaded area shows the standard deviation over five repetitions per hidden layer size. Variation was quite small for repeated learning experiments, suggesting that training time was sufficient for a good comparison.

In case of the front-to-middle-leg mapping (Figure 11A), small hidden layers introduced a bottleneck into the network, such that the performance of these networks was worse than linear regression. Only when four or more hidden neurons were used, the network performance improved continuously with increasing hidden layer size. Beyond a hidden layer size of 32 neurons, the MSE decreased only little, suggesting that additional complexity of larger ANNs would not pay off in terms of accuracy. Finally, the similar MSE curves for front-to-back and back-to-front projections suggested that a pair of reciprocal ANNs would work equally well in both directions. The top right subfigure adds the training time as the second independent axis, revealing that the networks converged nicely and that the training time of 5,000 epochs is sufficient for convergence, even for the more complex models. In general, the mapping problem appears sufficiently simple for continuous improvement with increasing model complexity

Results on a test data-set for the middle-to-hind-leg mapping were similar to those for the front-to-middle-leg mapping in that a minimum of four hidden neurons were necessary to achieve better performance than a linear regression. Also, the mapping accuracy improved continuously (the MSE decreased) with hidden layer size and the learning curve (top right insert) was equally smooth and monotonously decreasing as before. However, two results differed for the two mappings. First, near-optimal accuracy for the ANN with 32 hidden neurons was approximately tenfold higher for the middle-to-hind-leg mapping (Figure 11B) than for the front-to-middle-leg mapping, reaching a root mean squared error below half a degree. The second difference concerned the difference in mapping accuracy for the two directions, the back-to-front mapping reaching the level of mapping accuracy of the front-to-middle leg mappings only. To analyse this further, we turned toward the data for the right affordance volumes (lower right inserts in Figures 11A,B). For those examples, the difference between front-to-middle-leg and middle-to-hind-leg mappings were less pronounced than for the corresponding left leg pairs.

Since already small to medium hidden layers proved to be sufficient for a good approximation of the mapping, especially in the case of the affordance volumes of middle and hind legs, we wondered whether another kind of ANN architecture could work equally well with even less neurons. This is because the number of 32 neurons in the hidden layer was still high compared to possible candidate neural structures in an insect. Therefore, we further extended the model by direct skip connections from joint angle inputs to target outputs. Our results showed that skip connections may introduce a significant improvement in the case of very small hidden layers which had previously introduced a bottleneck effect (see Supplementary Figure 1, where the MSE for an ANN with 2 hidden neurons was as lows as 7.4 compared to 10.0 for the regression approach). However, the positive effect of skip connections vanished for more complex models. Probably, this was because skip connections only introduced a small number of additional connections compared to the growing number of connections toward and from the hidden layer. We conclude that for each affordance volume of ipsilateral leg pairs, very small feed-forward neural networks can achieve a better mapping performance than a linear regression, and that very high accuracy may be achieved with hidden layer sizes around 32 neurons.

## Discussion

Using the stick insect as an example, our study proposes a method to delineate distinct, behaviourally relevant spatial volumes in the near-rage environment of the insect body, based on experimental data. The first of these volumes is equivalent to what is typically referred to as peripersonal space in humans and comprises the action volumes of all eight limbs of the insect (six legs and two antennae; Figure 4). Essentially, our method assumes that this volume is defined by motor activity, as it is the volume traversed by any kind of limb movement that is likely to be observed during the behavioral paradigms considered. Nevertheless, it is important to note that this volume is also a volume of distinct sensory activity in that any contact-induced sensory activity can only occur within reach of a limb. Therefore, the boundary of peripersonal space can be viewed as the boundary beyond which motor activity cannot coincide with mechanosensory cues of physical contact. As a corollary, peripersonal space must be represented by distinct patterns of neural activity within the somatosensory and motor system of an insect. The behavioral relevance of the second volume—the affordance space—is given by the spatial correspondence of contact points that may be reached by two or more limb postures, either sequentially or simultaneously. The affordance space was therefore defined as that part of peripersonal space that fulfills the following two criteria: (i) it must be traversable by at least two different limbs (as judged by overlap of two action volumes in Figure 5) and (ii) the part of the limb that traverses must be likely to experience physical contact in natural behavior (e.g., the distal third of the limb, as justified by Figures 6, 7).

In our study, the first of these criteria (overlap) was applied only for ipsilateral pairs of limbs. Contralateral overlap was not considered because there are no dedicated experimental studies on bilateral spatial coordination of limbs in insects that could possibly contribute sufficient experimental data. Owing to the data-driven calculation of affordance space, the applicability of our method critically depends on the suitability of available motion capture data. In our case, the choice of climbing and searching paradigms would have been appropriate to estimate contralateral overlap for front legs and antennae, but much less for middle and hind legs. Since all experimental setups (Figure 2) were based on a horizontal walkway, the likelihood of middle and hind legs to cross the sagittal plane was limited to very rare and brief episodes of cyclic searching movements if a swing movement missed the obstacle. Future experimental studies will be needed to address contralateral coordinate transfer. Likely suitable behavioral paradigms would be gap-crossing with increased likelihood of searching movements of middle and hind legs (e.g., see Durr, 2001), or climbing along narrow substrates (e.g., see Cruse et al., 2009).

### The Role of Contact

Contact events are of particular relevance to both peripersonal space and affordance space. This is justified by the certainty of the sensory event of physical contact, and by the immediate behavioral relevance. An important factor contributing to the certainty of contact cues is “resisted movement” that is known to cause shear forces that stimulate strain-sensing campaniform sensilla in the cuticle (Zill et al., 2012). A second factor is the experience of coincident motor and sensory activity through proprioceptive postural feedback, strain-induced feedback, and, potentially, further sensory activity caused by exteroception of contact cues (touch). The immediate behavioral relevance of contact cues is related to the presence of an external object within the action range of the body.

So far, our considerations on likely contact locations (criterion 2 for affordance space) are restricted to antennae (Figure 6) and front legs (Figure 7). Future studies will need to record contact locations at the other limb pairs, in order to test whether the results on contact locations on front legs can be transferred to middle and hind legs. Moreover, it could be intriguing to distinguish distinct movement types subsequent to limb contacts, for example re-positioning in case of inappropriate foothold, or retraction in case of proximal contact sites. Although there is some evidence that physically interrupted swing movements of walking stick insects always follow a default retraction-levation-flexion response (Ebeling and Dürr, 2006), existing studies did not control for the contact site along the limb. To date, contact-induced limb-movements have been described qualitatively in stick insects (e.g., Bläsing and Cruse, 2004; Theunissen and Dürr, 2013) and cockroaches (e.g., Ritzmann et al., 2000), but were not related to preceding contact locations.

Another limitation of the existing experimental data concerns the restriction to initial contacts. Potentially, this leads to underestimating the likelihood of proximal contacts. In case of the antennae, this can be expected from results of Krause and Dürr (2012), who studied antennal tactile sampling behavior of stick insects that climbed a stair of varying height. That study categorized antennal contact locations on the obstacle as “along the frontal wall” and “on the upper edge” of the stair and found that the prior category occurred predominantly near the tip (a region corresponding very well to that shown in Figure 6), whereas the latter category occurred predominantly along more proximal parts of the flagellum. In case of the legs, the relatively small difference of the affordance volume sizes for action volumes vs. contact volumes (Figure 9) suggests that the inclusion of more proximal contact locations would have little effect on the affordance volumes of leg pairs. In case of the antenna, however, the effect would be much stronger, as the gap between the head and the antenna-front-leg affordance volume would shrink.

### Modeling Affordance Space

Since our definition of affordance space is based on the transfer of spatial information among limbs, we probed the computational properties of the mappings within pair-wise affordance volumes with Artificial Neural Networks (ANN) of differing complexity (Figure 10). ANNs were trained to map the posture of a sender leg to the corresponding posture of a neighboring receiver leg with equal foot position. The output of the ANN can be viewed as a target posture that can be used to control the movement of the receiver leg. A first model for such leg targeting behavior was introduced by Dean (1990) to simulate the spatial coordination of lift-off and touch-down locations of ipsilateral leg pairs in stick insects. This model was later included as the so-called *target net* in *Walknet*. *Walknet* is a behavior-based, distributed ANN control model of multi-legged locomotion in animals and walking robots (Cruse et al., 1995; Schmitz et al., 2008; for the most recent version, see Schilling et al., 2013). In *Walknet*, spatial control of foot position, i.e., targeting, was originally realized by a simple feed-forward neural network that only consisted of one hidden layer with three hidden neurons. In later versions of *Walknet*, the *target net* also included skip connections (Cruse et al., 1998; Dean et al., 1999), as tested by the present study (Supplementary Figure 1). Already this small network could simulate spatial targeting behavior of an insect walking on a plane. The original target net was analyzed only qualitatively and postures were restricted to much smaller working ranges (i.e., action volumes). Moreover, the resulting walking behavior of *Walknet* was quite regular. In contrast to the mentioned studies on *Walknet*, we provide a quantitative analysis of the complexity of that part of this control network that deals with spatial inter-limb coordination (the *target net*). Major differences of our present model and *target net* are (i) the considerably larger action volumes of the limbs, owing to the much larger behavioral variability, (ii) the consideration of all three spatial dimensions, and (iii) the systematic and quantitative evaluation of mapping accuracy as a function of network complexity. Our results show that the ANN structure used by Dean (1990) was insufficient to achieve an accurate mapping for our experimentally derived affordance volumes (being in the bottleneck range of Figure 11, with inferior accuracy than a linear regression). However, highly accurate mappings can be learnt with more hidden neurons. For the middle-to-hind-leg mappings, appropriate network structures were still small. As for the front-to-middle-leg mappings, the affordance volume was much larger than for middle-to-hind-leg mappings, equal accuracy required more neurons. However, in both cases accuracy was very high for moderately sized network topologies (between 8 and 32 hidden neurons). These numbers are in the range of what would be plausible for a physiological neural network realized in the insect. For example, von Uckermann and Büschges (2009) described twelve non-spiking premotor interneurons for the mesothoracic ganglion of the stick insect, all of which are candidates for being involved in the local control of leg movements. As mentioned, Dean et al. (1999) employed skip connections for their target network approach to improve accuracy. Our results confirm that skip connections can lead to an improvement of targeting accuracy for small networks (less than 4 hidden neurons; Supplementary Figure 1), but not for larger networks (which would be required for high accuracy). A next step could be to extend the ANN toward more hidden layers and deeper architectures. However, this should be related to known properties of the neural organization of the insect sensorimotor system. In insects, both the terminal arborisations of leg proprioceptor afferents, and the dendrites of motoneurons of a leg are always confined to the ganglion of the same thorax segment. As a consequence, the transfer of limb posture information from one leg to another requires at least one layer of intersegmental neurons that mediates the afferent input from one segment to the efferent output neurons in the next segment. Intersegmental neurons that mediate postural information have been described for stick insects (Brunn and Dean, 1994). However, whether these intersegmental neurons connect to motor neurons directly (corresponding to one hidden layer), or to local premotor interneurons (corresponding to two hidden layers) or both (two hidden layers with skip connections that shortcut hidden layer 2) is unknown. In case of the antenna-to-front-leg mapping, skip connections would be plausible because proprioceptive afferents from antennal joints have collateral projections to the brain and to the suboesophageal (gnathal) ganglion (e.g., Goldammer and Dürr, 2018). However, these skip connections would not connect to the output layer (motoneurons of the front leg), but to at least one further hidden layer.

With regard to the asymmetry of backward and forward projections described in Figure 11, it appears that the sampled data for the left middle-to-hind-leg mapping (and to limited extent for the right middle-to-hind-leg mapping as well) contains some underlying regularity which makes it easier to learn the mapping in one direction than in the other, opposite direction. A possible explanation for this could be related to nested trigonometric functions involved in the mapping of limb postures, where small changes in the input or output ranges could both favor or prevent successful inversion. For example, consider approximating a sine function: when considering the range around zero only, this function can be linearly approximated and inverted. As yet, the function values around π/2 are all close to one and inversion is impossible.

We conclude that ANNs provide a good model for the affordance space defined here. The model could account for information transfer about footholds among limbs. In humans, Magosso et al. (2010) realized a model for—what they call more generally—peripersonal space through artificial neural networks. Their work is comparable, as it is based on trained mappings between different spatial representations that relate locations of limbs among each other. As a key difference, their work focuses on visuo-tactile representations and takes inspiration from human cortical representations, whereas our work aims at simpler models. But while the function of the sub-components is comparable, they further show how these can be interconnected, thus giving rise to a body model. In another example, Braud et al. (2018) introduced an anticipatory model for grasping that aims to learn the combination of actions and their associated perceptual effects. This is then exploited for motor planning by a form of mental simulation. Like our study, Braud et al. focus on behavioral relevance by directly relating sensory information to the action capabilities of the system. In general, body model representations are used widely in robotics (e.g., Lallee and Dominey, 2013, for review see Schillaci et al., 2016). They are assumed to be quite flexible in both humans and animals and allow for cognitive abilities such as movement planning. So far, most existing models in robotics deal with visuo-tactile coordination and the control of reaching or grasping movements (Hoffmann et al., 2010). These approaches could benefit from including further modalities.

The basic mappings as used to model our affordance space may be arranged to constitute a body model too, e.g., by application of the “Mean of Multiple Computations” (MMC) principle as done by Schilling (2011). The MMC principle breaks down the complexity of a sensorimotor system into multiple local relationships, each one of which expressing a relatively simple transformation. The mappings analyzed here are examples of local relationships for pairs of parallel kinematic chains. As such, they could be integrated into an MMC model of an entire insect body or of any other body scheme including multiple limbs.

## Conclusion

In summary, we argue that invertebrates have at least two internal representations of space: one far-range representation of the space “beyond reach” that is required for orientation and navigation behavior (e.g., see Heinze and Pfeiffer, 2018, and the corresponding special issue), and one near-range representation of the space “within reach” that is required for spatial coordination of limbs. With regard to the latter, we demonstrate that the joint action ranges of two neighboring legs are almost equivalent to the overlap regions in which physical contact with the environment is likely to occur. We call these joint action ranges affordance volumes. Finally, we propose basic computational elements that relate the posture of one limb to that of another and, thus, serve as models for spatial inter-limb coordination in general. Since each one of these elements is experimentally grounded in a database of natural movement sequences, they model behaviourally relevant coordinate transformations within the natural action range of an insect. Owing to the directedness of the transformations, i.e., the property that one (sender) leg informs another (receiver) leg how to reach the same foot position, they implement affordances for spatially coordinated limb movements. We argue that these affordances for spatial inter-limb coordination define a subspace of peripersonal space that is essential for any behavior that requires spatial control of footfall patterns (in climbing this may be vital) or bimanual coordination, Given the ubiquity of spatial inter-limb coordination behavior in animals, this affordance space must be a fundamental property of motor systems with multiple limbs.

## Author Contributions

VD conceived the study and did experimental analysis, MS did neural network simulations, VD and MS discussed the results, prepared the figures, and wrote the paper.

### Conflict of Interest Statement

The authors declare that the research was conducted in the absence of any commercial or financial relationships that could be construed as a potential conflict of interest.
