# Supplementary material for: Transfer of Spatial Contact Information Among Limbs and the Notion of Peripersonal Space in Insects
Source: Front Comput Neurosci. 2018 Dec 18;12:101. doi: 10.3389/fncom.2018.00101 (PMC6305554; doi:10.3389/fncom.2018.00101)
Supplement: Supplementary file 1 [file Data_Sheet_1.pdf]

Transfer of spatial contact information among limbs and the notion of peripersonal space in insects

Volker Dürr and Malte Schilling

Supplementary Material

Supplementary Table 1: Threshold densities and corresponding fraction of the total density for the 3x4 combinations of volume type and limb. Numbers in the first row indicate the threshold density equivalent to 1% of the maximum density for each type of volume. Left and right numbers correspond to [left limb; right limb]. The numbers of the second rows list the fraction of summed volume density comprised when applying the threshold density noted above.

	**Tip**	**Contact**	**Action**
**Antenna**	[2.8, 3.8]<br>[97.0, 96.6]	[1.0, 1.4]<br>[98.0, 97.9]	[1.0, 1.1]<br>[97.5, 97.8]
**Front leg**	[2.3, 3.1]<br>[96.4, 95.3]	[1.6, 1.7]<br>[96.2, 96.2]	[2.1, 1.9]<br>[95.7, 96.3]
**Middle leg**	[4.0, 4.8]<br>[98.7, 98.3]	[2.8, 2.5]<br>[98.5, 98.6]	[3.5, 3.6]<br>[98.3, 98.2]
**Hind leg**	[6.7, 5.1]<br>[96.6, 97.5]	[3.9, 3.0]<br>[97.0, 97.7]	[3.6, 3.4]<br>[97.5, 97.6]

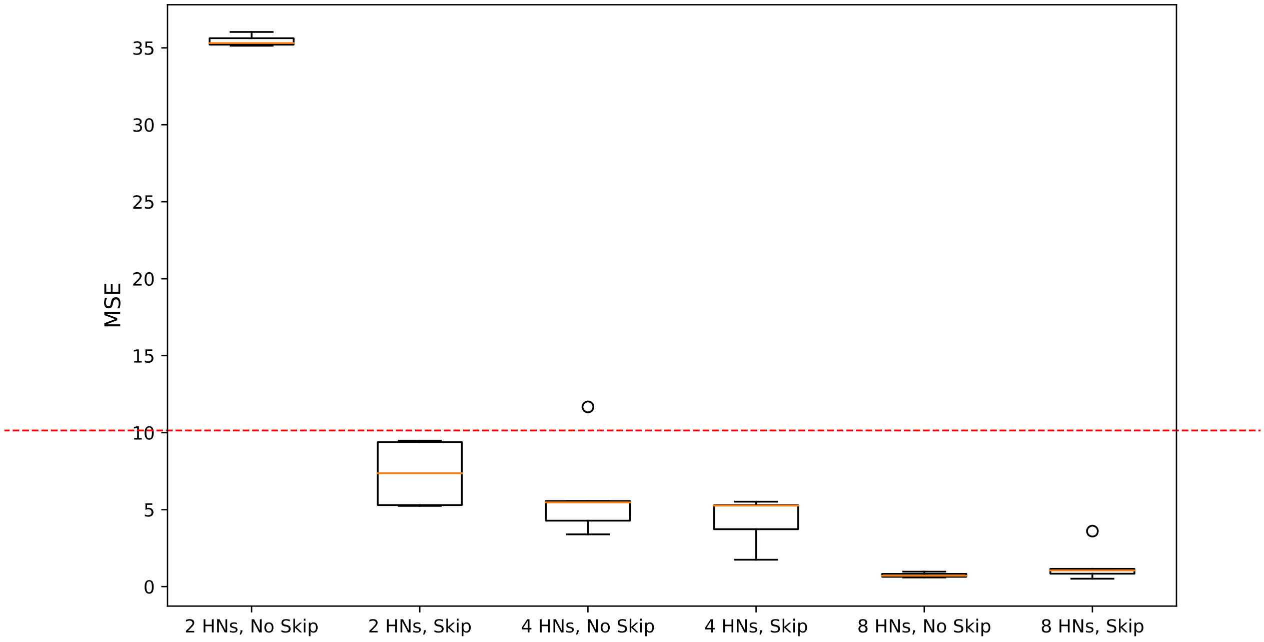

Supplementary Figure 1: Skip connections can improve small networks only. Comparison of small three-layered ANNs with and without skip connections. Box-whisker plots show the accuracy after 5000 training episodes for the affordance space between left middle and hind legs and the front-to-back projection. With two hidden neurons only, additional skip connections significantly improve performance. With larger hidden layers, skip connections introduce little or no improvement. The relatively large variation for small hidden layers also points out that these small network structures are more prone to converge towards local minima, thus introducing more variation among solutions.
